# Enhancing droplet-based single-nucleus RNA-seq resolution using the semi-supervised machine learning classifier DIEM

**DOI:** 10.1038/s41598-020-67513-5

**Published:** 2020-07-03

**Authors:** Marcus Alvarez, Elior Rahmani, Brandon Jew, Kristina M. Garske, Zong Miao, Jihane N. Benhammou, Chun Jimmie Ye, Joseph R. Pisegna, Kirsi H. Pietiläinen, Eran Halperin, Päivi Pajukanta

**Affiliations:** 1 Department of Human Genetics, David Geffen School of Medicine at UCLA, Los Angeles, CA 90095 USA; 2 Department of Computer Science, School of Engineering, UCLA, Los Angeles, CA 90095 USA; 3 Bioinformatics Interdepartmental Program, UCLA, Los Angeles, CA USA; 4 Department of Epidemiology and Biostatistics, Department of Bioengineering and Therapeutic Sciences, Institute for Human Genetics, UCSF, San Francisco, USA; 5 Vache and Tamar Manoukian Division of Digestive Diseases, UCLA, Los Angeles, CA USA; 6 Obesity Research Unit, Research Programs Unit, Diabetes and Obesity, University of Helsinki, Biomedicum Helsinki, Helsinki, Finland; 7 Obesity Center, Endocrinology, Abdominal Center, Helsinki University Central Hospital and University of Helsinki, Helsinki, Finland; 8 Department of Human Genetics, Institute for Precision Health, David Geffen School of Medicine at UCLA, Gonda Center, Room 6335B, 695 Charles E. Young Drive South, Los Angeles, CA 90095-7088 USA; 9 Department of Anesthesiology, UCLA Health, Los Angeles, CA 90095 USA; 10 Department of Computational Medicine, School of Medicine, UCLA, Los Angeles, CA 90095 USA; 11 Institute for Precision Health, School of Medicine, UCLA, Los Angeles, CA 90095 USA

**Keywords:** Functional clustering, Machine learning, Gene expression profiling, Transcriptomics

## Abstract

Single-nucleus RNA sequencing (snRNA-seq) measures gene expression in individual nuclei instead of cells, allowing for unbiased cell type characterization in solid tissues. We observe that snRNA-seq is commonly subject to contamination by high amounts of ambient RNA, which can lead to biased downstream analyses, such as identification of spurious cell types if overlooked. We present a novel approach to quantify contamination and filter droplets in snRNA-seq experiments, called Debris Identification using Expectation Maximization (DIEM). Our likelihood-based approach models the gene expression distribution of debris and cell types, which are estimated using EM. We evaluated DIEM using three snRNA-seq data sets: (1) human differentiating preadipocytes in vitro, (2) fresh mouse brain tissue, and (3) human frozen adipose tissue (AT) from six individuals. All three data sets showed evidence of extranuclear RNA contamination, and we observed that existing methods fail to account for contaminated droplets and led to spurious cell types. When compared to filtering using these state of the art methods, DIEM better removed droplets containing high levels of extranuclear RNA and led to higher quality clusters. Although DIEM was designed for snRNA-seq, our clustering strategy also successfully filtered single-cell RNA-seq data. To conclude, our novel method DIEM removes debris-contaminated droplets from single-cell-based data fast and effectively, leading to cleaner downstream analysis. Our code is freely available for use at https://github.com/marcalva/diem.

## Introduction

Single-cell RNA sequencing (scRNA-seq) has grown considerably in use over the previous decade and permitted a transcriptomic view into the composition of heterogeneous mixtures of cells^1,2^. Recent advances in droplet-based microfluidics have created a high-throughput opportunity to assay single cells by scaling up previous well-based technologies to tens to hundreds of thousands of cells^3^. Single-nucleus RNA sequencing (snRNA-seq), where nuclei are used instead of cells, has allowed the critical extension of single-cell based technologies to solid tissues where isolation and suspension of individual cells is difficult or impossible^4^. For example, snRNA-seq has been used successfully to identify cell types in the brain^5^. Another practically important application of sequencing nuclei is identifying cell types in frozen tissue, from which it is often not feasible to isolate intact cells, whereas nuclei can still be successfully isolated^6^.

Droplet-based snRNA-seq encapsulates individual nuclei into a water-in-oil emulsion that contains reagents for generating cDNA and ligating droplet-specific oligonucleotide barcodes. After library construction and sequencing, the mapped reads can be assigned to droplets of origin. The input nuclei suspension is prepared so that all reads associated with one barcode originate from one nucleus. However, RNA originating from lysed cellular components (such as the cytoplasm) or from outside the cell can become encapsulated in droplets as well. Since these reads have the same barcode, contaminated RNA cannot be readily distinguished from nuclear RNA. To apply snRNA-seq to tissues, homogenization of the tissue is usually required to break apart the extracellular matrix and release nuclei from cells^4^. This can release higher amounts of debris and lead to more background RNA contamination^7^. This contamination of droplets with extranuclear RNA can lead to a biased increase in expression of these genes. Using mitochondrial RNA, we show that this results in clusters driven by background RNA, as well as contamination of clusters representing true cell types. As droplet-based snRNA-seq is increasingly applied to various solid tissues, there is an urgent need to accurately filter contaminated droplets.

A common practice to distinguish cell/nuclei- vs. background-containing barcodes relies on removing droplets below a hard cutoff of the number of reads, unique molecular indexes (UMI), or genes detected in a droplet^3,8–11^. This ad hoc cutoff is typically set by ranking barcodes by their total UMI counts and visually selecting a knee point, where a steep dropoff in counts occurs^3,12^. Droplets with higher counts are expected to contain cells or nuclei, whereas droplets with lower counts are expected to contain ambient RNA. However, a clear separation between the two may not occur, especially if the amount of debris is high and the droplet RNA content is low, as we show is the case with frozen solid tissues. Additionally, an ad hoc cutoff of the percent of reads originating from the mitochondria (a measure of extranuclear contamination) can help to filter droplets^12^. Again, the choice of a cutoff may be arbitrary or unclear. The recent method EmptyDrops^12^ addresses this filtering issue for scRNA-seq by estimating a Dirichlet-Multinomial distribution of the ambient RNA. It then removes droplets by testing if their expression profile deviates significantly from the ambient profile using a Monte Carlo approach^12^. However, while this works for single-cell, we show that these methods underperform when applied to snRNA-seq.

Here we show that, in snRNA-seq, using a hard cutoff to remove droplets can result in a substantial loss of nuclear droplets and inclusion of debris droplets. Importantly, we demonstrate that including these contaminated droplets can lead to spurious clustering and false positive cell types. To overcome this, we built a fast filtering pipeline that uses a likelihood-based approach to model debris and cell type RNA distributions with a multinomial distribution. The parameters of the model are inferred using semi-supervised EM^13,14^, where all droplets below a hard count threshold are fixed as debris. Then, the droplets are scored based on their expression of genes enriched in the debris set. This multinomial-based clustering approach has been successfully applied to the information retrieval and text mining fields^15^. Similar to reads, word occurrences in a document can be modeled with a multinomial distribution, and documents can belong to separate topics, leading to a mixture model.

We developed this pipeline into an approach, termed Debris Identification using EM (DIEM), which robustly removes background droplets from both scRNA-seq and snRNA-seq data. In contrast to hard count and EmptyDrops filtering, DIEM accurately models debris and cell types and can quantify the amount of contamination in individual droplets. This resulted in more accurate filtering and higher quality clustering of snRNA-seq data, particularly when applied to frozen tissue. We also found that DIEM can effectively filter scRNA-seq data.

## Results

### snRNA-seq produces clusters driven by high amounts of background RNA contamination

**Figure 1:**
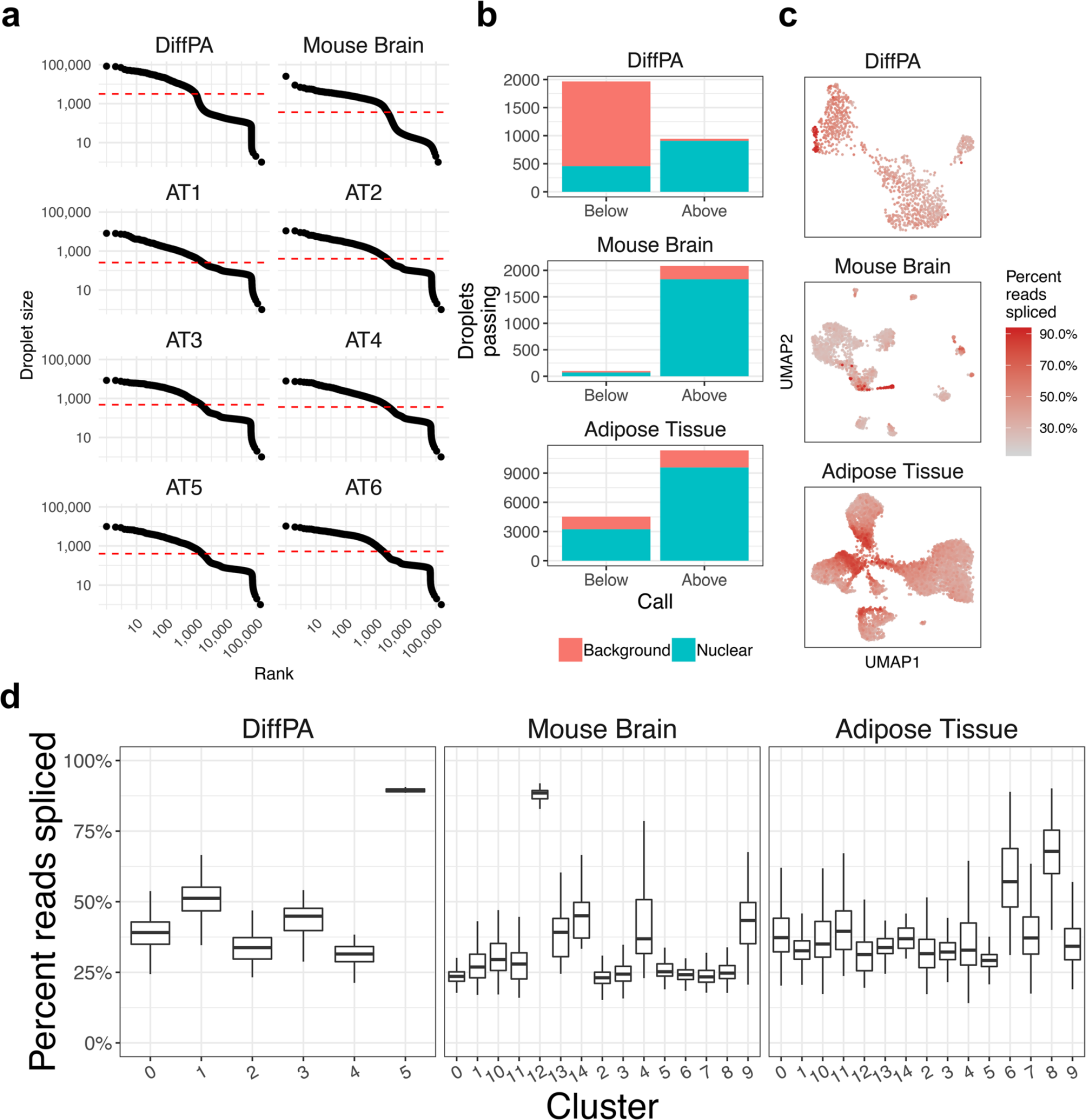
Applying a hard count threshold fails to remove droplets contaminated with background RNA in snRNA-seq. (**a**) Barcode-rank plots showing the droplet size (the total number of UMI read counts) of each droplet in descending order for the differentiating preadipocytes (DiffPA), mouse brain, and six human frozen adipose tissue (AT) snRNA-seq samples. The dotted red line indicates the quantile-based threshold. (**b**) The number of droplets above and below the quantile-based hard-count threshold is shown. The height of the red bar indicates the number of background droplets in the category indicated in the x-axis, while the height of the blue bar indicates the number of nuclear droplets. Background and nuclear droplets are defined using the percent spliced reads. Ideally, all nuclear droplets would occur above the threshold and all background droplets would occur below. (**c**) UMAP^33^ visualization of droplets in each of the three data sets with droplets colored by the percent of reads spliced. (**d**) The droplets above the quantile threshold were clustered using Seurat^20^. The x-axis shows the clusters, and the y-axis shows the distribution of the percent of reads spliced for each cluster. Background droplets with a high percent of reads spliced tend to cluster together.

Isolation of nuclei for snRNA-seq relies on lysis of the cell membrane, releasing cytoplasmic RNA, in addition to cell-free RNA, into the solution. This extranuclear RNA can become encapsulated into droplets, with or without nuclei, and lead to biases in downstream analysis; particularly, it may lead to spurious or contaminated cell-types in downstream clustering. We evaluated the extent of contamination and its effect on clustering in three distinct snRNA-seq data sets: 1. in vitro differentiating human preadipocytes (DiffPA) (n = 1), 2. freshly dissected mouse brain tissue (n = 1), and 3. frozen human subcutaneous adipose tissue (AT) (n = 6). We initially ran a clustering analysis in the three data sets by filtering out droplets with a hard-count threshold^3,8–11^. This threshold can be selected manually, as the knee point^3^, or by dividing the total count of the 99% quantile of expected cells by 10^16^. Since we observed that the knee point could not be reliably estimated or was not evident in the AT samples (Fig. 1a), we used the quantile-based threshold for further analyses.

To assess levels of extranuclear RNA contamination, we primarily used the percentage of reads that are spliced in a droplet. The poly-T capture probes used in drop-seq ^3^ and the 10X platform can also hybridize to adenosine tracts present in introns, allowing for quantification of unspliced pre-mRNAs^17^. We expected that a higher percent of cytoplasmic ambient RNA would be spliced in comparison to nuclear RNA, and thus contaminated droplets would have a higher proportion of spliced reads. We found that in all three data sets, the percent of spliced reads correlated negatively with total UMI counts (Fig. S1a). Furthermore, we found that the percentage of reads spliced generally showed a bimodal distribution, with nuclear and background droplets centered below and above roughly 50%, respectively (Fig. S1b). For each of the 8 experiments, we calculated a midpoint to separate the nuclear and background distributions (see “Methods”). This was performed independently for each experiment as they exhibited distinct distributions (Fig. S1b). To evaluate clusters, we specified those with a mean percent of reads spliced of at least 50% as debris and classified those with less than 50% as cell types consistent with expressed marker genes, as we observed this was the average value across the experiments and that the 6 adipose tissue samples were combined. In addition to the percentage of reads spliced, we evaluated extranuclear contamination using the percentage of UMIs aligning to the mitochondria (MT%) and to the nuclear-localized lincRNA *MALAT1*^18^ (MALAT1%). We chose to incorporate mitochondrial RNA as a measure extranuclear RNA contamination because it is one of the only true sources of background RNA and is present in all snRNA-seq data sets. However, we note that other sources of extranuclear RNA can exist. Hemoglobin mRNA, which is predominantly expressed in erythrocytes, can also serve as another negative control for tissues where blood is present^19^. We also found that the percentage of reads spliced correlated positively with MT% and negatively with MALAT1% (Fig. S1c, d). As the percentage of spliced reads is more independent from gene expression, we primarily used this metric as an estimate of contamination within droplets.

To test whether a hard count threshold could effectively remove debris-contaminated nuclei, we investigated the relationship between total counts and the percent of reads spliced. We selected the hard count threshold for each of the 8 independent samples based on a quantile^16^ (Fig. 1a). We found that this threshold failed to remove all background droplets and incorrectly removed nuclear droplets (Fig. 1b, c). For example, in the DiffPA dataset, the quantile threshold correctly kept a large proportion of nuclear droplets (908 of 944) but incorrectly removed 457 droplets (Fig. 1b). Of the 11,331 passing droplets in the 6 adipose tissue samples, only 9,578 (84.5%) droplets were nuclear (Fig. 1b). We found that no single count threshold could effectively discriminate the nuclear and background droplets (Fig. S1a). We further investigated the downstream effect on clustering to see if there was any evidence of background RNA driving spurious clusters. In the DiffPA, mouse brain, and adipose tissue data sets, there were 2, 1, and 2 clusters that had a mean percent reads spliced greater than 50%, respectively (Fig. 1d). Additionally, we observed droplets with a high MT% and clusters that were enriched for mitochondrial RNA (Fig. S2). Overall, a hard count threshold failed to discriminate nucleus-containing droplets from debris droplets when using percent reads spliced and MT% to quantify contamination.

### Nuclear and debris droplets demonstrate distinct RNA profiles

**Figure 2:**
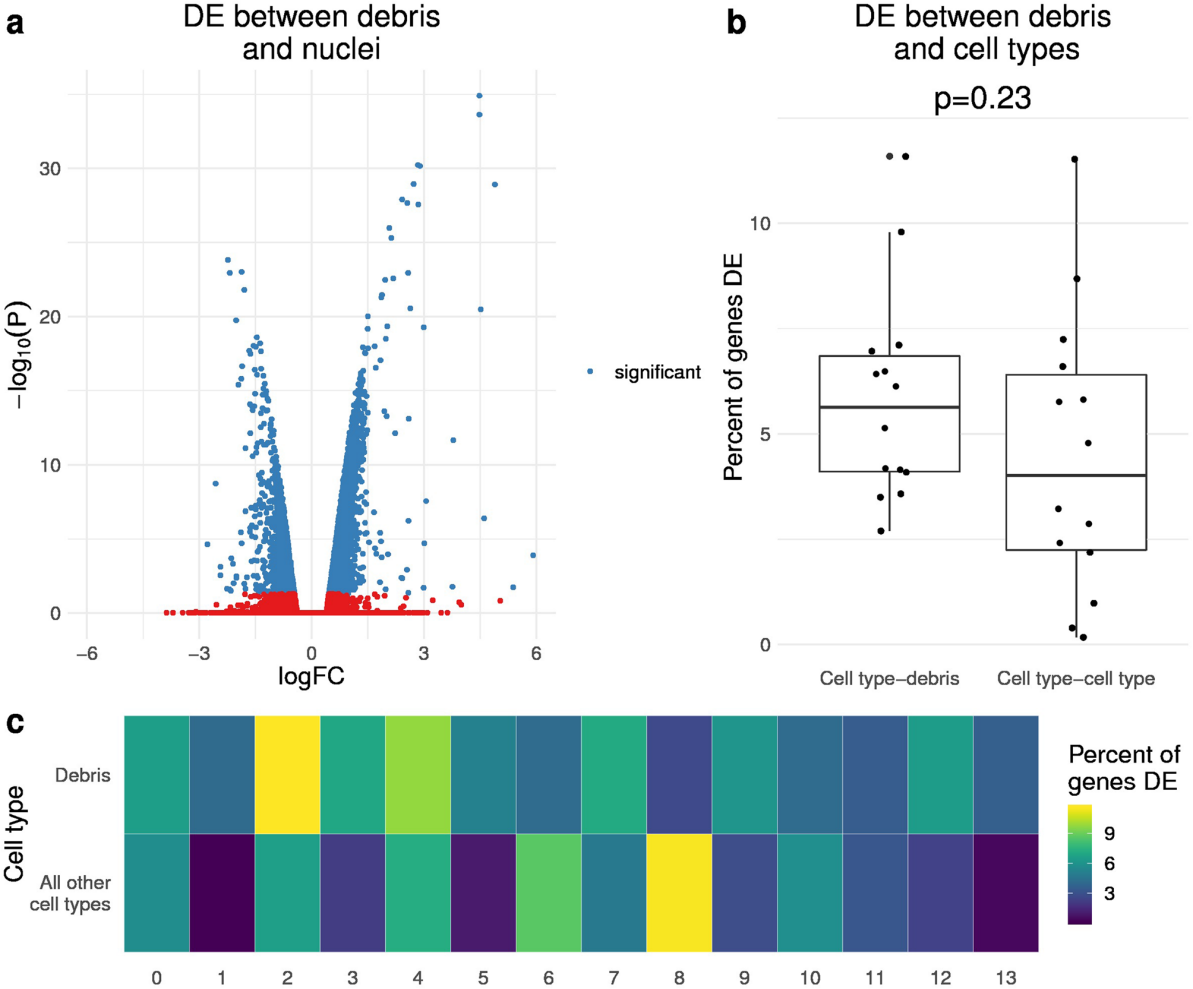
Debris-containing and nuclei-containing droplets show distinct gene expression profiles. (**a**) Differential expression (DE) between droplets with less than 100 UMI counts (debris) and greater than or equal to 100 UMI counts (nuclei) in the 6 human adipose tissue (AT) samples. The volcano plot shows the log fold change on the x-axis and negative log transformed p-value on the y-axis. The genes colored in blue are DE with a Bonferroni-corrected p-value < 0.05. A positive log fold change indicates over-expression in the debris group. (**b**, **c**) For each of the 14 cell types identified after clustering the quantile filtered droplets, we ran differential expression between the cell type and the debris group, or between the cell type and all other cell types in the combined adipose tissue data set. Cell types are estimated from clustering droplets that pass quantile-based filtering. A (**b**) box plot shows the percent of expressed genes that are DE (Bonferroni p < 0.05) between a cell type-debris pair, and a cell type-cell type pair. The p-value was calculated from a student’s t-test between cell type-debris percent and cell type-cell type percent. The (**c**) heatmap shows the percent of total genes expressed in the cell type (x-axis column) that are significantly differentially expressed between the debris droplets (first row) or droplets in all other cell types (second row). This shows that the DE between a cell type and the debris group is similar to the DE between different cell types.

Since the total UMI count in a droplet does not always distinguish nuclei from debris, we postulated that the expression profile of a droplet could be used to differentiate them if there were sufficient differences in RNA abundance between cell types and debris. Specifically, we hypothesized that there would be genes with sub-cellular localized RNA products that show differential abundance between droplets containing nuclear vs. ambient RNA. Thus, we evaluated the extent of differences between the debris and nuclear RNA profiles. We separated droplets into debris- and nuclear-enriched groups using a threshold of 100 total UMI counts. Although a large number of droplets above 100 UMI counts consist of debris and would lead to a loss of power, we use this threshold to ensure that no droplets below it contain nuclei. We evaluated the difference between the debris and nuclear RNA profiles by running a paired differential expression (DE) analysis in the six human AT samples. Of 19,934 genes detected, 3,417 (17.1%) were DE between the nuclear- and debris-enriched groups at a Bonferroni-adjusted p-value threshold of 0.05 (Fig. 2a). To see if these differences were preserved across the DiffPA, mouse brain, and six AT data sets, we correlated the nuclear vs. debris log fold changes of the genes in common. Among the 8,924 genes expressed in all three data sets, we found that all log fold changes were significantly correlated (p < 2.2 × 10^−16^) across all pairs (mean *R* = 0.56), with the human data sets showing the highest correlations (Fig .S3).

Since the nuclear-enriched group is not homogeneous, but rather originates from distinct cell types with different RNA distributions, we also looked at differences between the debris group and cell types. In addition, we compared the cell type-debris differences with the cell type-cell type differences. Using the six AT samples, we ran a paired DE analysis between the cell types and debris droplets (total UMI counts < 100). Among 14 debris-cell type pairs, the average percent of genes that are DE was 5.8% (Fig. 2b). We then compared this to the DE between a cell type and all other cell types. Among these 14 pairs, the average percent of genes DE between cell types was slightly lower at 4.5% (t-test p = 0.23; Fig. 2b, c). Overall, we found significant differences between debris and nuclei RNA profiles, and that the differences between debris and cell types were within the same order of magnitude as the cell type-cell type differences.

### Overview of a novel EM-based approach to cluster and remove debris droplets from snRNA-seq data

**Figure 3:**
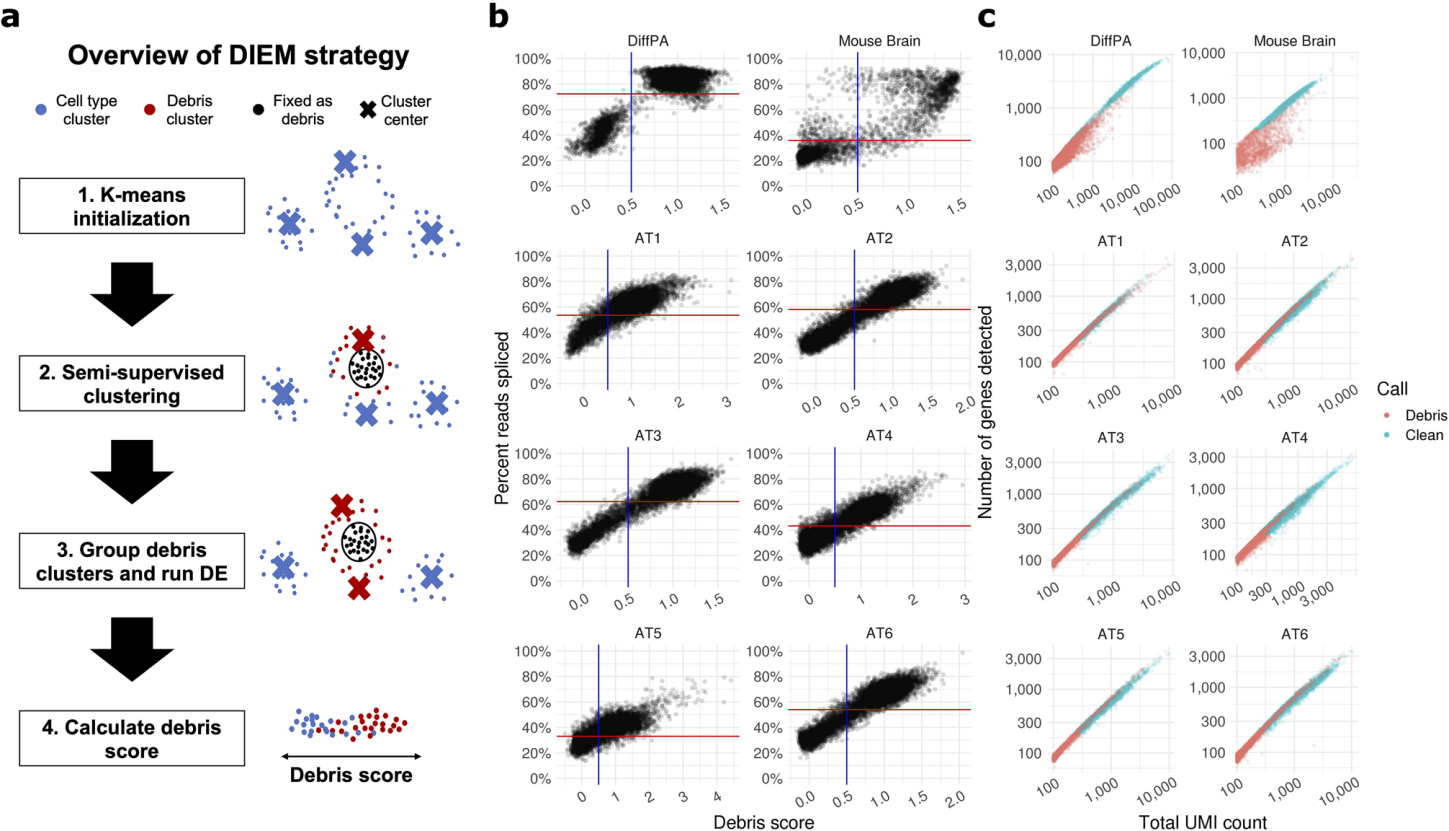
Debris scoring predicts background RNA contamination in snRNA-seq droplets. (**a**) Overview of DIEM approach to remove debris-contaminated droplets. Expectation Maximization (EM) is used to estimate the parameters of a multinomial mixture model consisting of debris and cell type groups. The label assignments of droplets below a pre-specified threshold (100 total counts) are fixed to the debris group, while the test set droplets above this rank are allowed to change group membership. The mixture model is initialized by running k-means. After parameter estimation, droplets are grouped into the debris cluster(s) or cell type clusters based on their posterior probabilities. Debris scores are calculated for each droplet by summing the normalized expression of debris-enriched genes, which are specified by differential expression between the debris and cell type clusters. Droplets can be filtered based on their cluster assignment or on their debris score. (**b**) The debris score of a droplet and the percent of reads spliced exhibit a significant correlation in the differentiating preadipocytes (DiffPA), mouse brain, and human frozen adipose tissue (AT) data sets (mean *R* = 0.89). The horizontal red line indicates the sample-specific midpoint that separates nuclear and background droplets. The vertical blue line indicates the threshold cutoff of 0.5 we used, where droplets with a debris score less than 0.5 are classified as clean. **c,** Scatterplots of droplets from snRNA-seq of the DiffPA, mouse brain, and AT data sets, with total unique molecular index (UMI) counts on the x-axis and total number of genes detected on the y-axis. Droplets are colored by the DIEM classification. Those in red are removed as debris while the blue droplets are kept as nuclei.

Since we observed differences in RNA abundance between cell types and debris, we developed an approach to remove debris-containing droplets based on the distribution of read counts. Our approach assigns individual debris scores to filter out droplets. We first cluster droplets using a multinomial mixture model**.** To estimate the parameters of the mixture model, we run semi-supervised expectation maximization^13,14^ by fixing droplets that fall below a threshold of 100 counts as debris. The majority of these droplets are assumed to contain ambient RNA, and thus we leverage this feature by fixing the labels throughout EM. After fitting the model, we assign droplets to clusters based on their posterior probability. Then, droplets are scored based on their expression of genes enriched in the debris set. DIEM then filters out droplets based on their individual scores. Figure 3a shows an overview of this model. We termed this method Debris Identification using Expectation Maximization (DIEM). We ran DIEM on the DiffPA, mouse brain, and six AT sample and compared our approach with the quantile-based method and the EmptyDrops method in the DropletUtils package^12^.

Although debris scores are used to filter out individual droplets, we run clustering to better initialize the debris and cell type groups. Droplets are clustered using a multinomial mixture model and the parameters are fit using semi-supervised EM. To initialize the EM, we run k-means with a pre-specified number of cell types k. After the initialization, semi-supervised EM estimates the parameters of the multinomial mixture model while fixing the labels of the low-count droplets to the debris cluster. The mixture model consists of k + 1 clusters corresponding to the debris cluster along with the cell type clusters initialized by k-means (Fig. 3a). Here, we set k to 20 for all experiments, although we noticed robust results across a range of k greater than 1 to 50 (Fig. S4). While it is possible to remove droplets that have high posterior probability of belonging to the debris cluster, we noticed that some of the cell type clusters produced by the mixture model contained contaminated droplets with high a percent of reads spliced in the snRNA-seq data sets (Fig. S5). Since only removing the debris cluster would fail to account for this, we developed an approach to estimate contamination in individual droplets instead (see “Methods”). Briefly, DIEM runs differential expression between the droplets in the debris and cell type clusters and calculates a debris score based on the debris-enriched genes. We found that this debris score correlated highly with the percent of spliced reads in all 8 independent snRNA-seq experiments (mean Pearson *R* = 0.89; Fig. 3b). To filter our droplets, we use a threshold *t* where those with a score above this value are removed. We investigated the effect of varying *t* from 0 to 1. As expected, the number of passing droplets increased with *t* (Fig. S6). However, the proportion of background droplets and contamination in the kept droplets also increased as *t* was increased (Fig. S6). We therefore set *t* to 0.5 for all experiments in the manuscript. Intuitively, this value represents the threshold that lies between the least contaminated cluster and the debris cluster.

The incorporation of clusters should result in a more realistic model of the snRNA-seq data. DIEM directly models debris and cell type clusters to more accurately specify the debris and cell type droplets for calculating the debris score. We asked whether the clusters identified by DIEM corresponded to valid biological cell types. DIEM identified 3 major cell types in the DiffPA, consisting of preadipocyte-like, fibroblast-like, and adipocyte cells (Fig. S7a). In the adipose tissue data sets, we found that the up-regulated cluster markers corresponded to the known major cell types in adipose, including immune, endothelial, fibroblast, and adipocyte cell-types (Fig. S7b). We then compared the DIEM clusters to those identified by the established method Seurat^20^ We found that the Seurat clusters generally overlapped with the DIEM clusters (mean percent overlap 73.0% across the 8 independent samples; Fig. S7c). Together, these results suggest that DIEM accurately identifies cell types and can leverage this cell-type heterogeneity to filter debris droplets.

### DIEM filtering results in a higher proportion of nuclear droplets and less contaminated clusters in snRNA-seq

**Figure 4:**
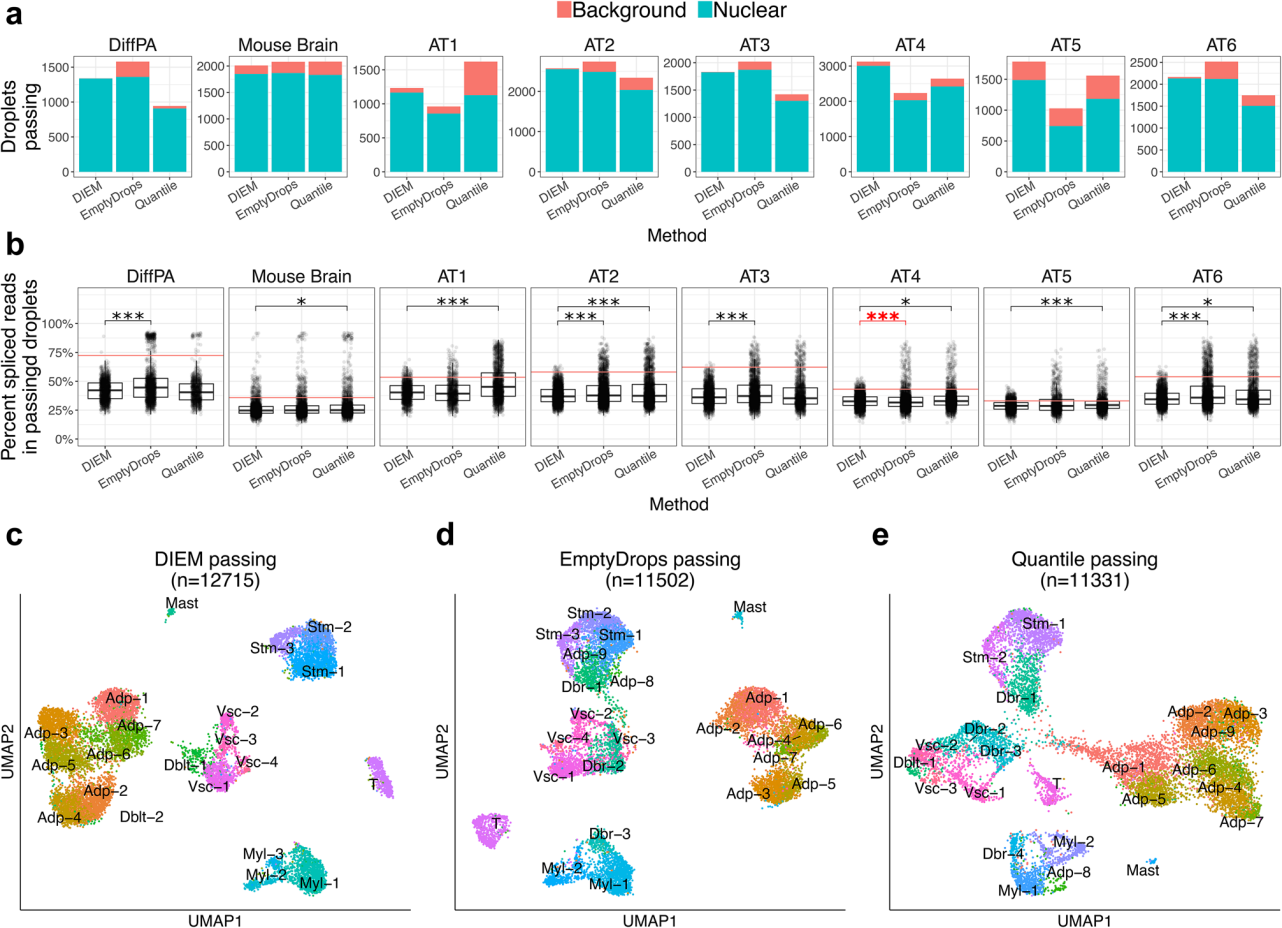
DIEM filtering keeps an increased number and proportion of nuclear droplets in snRNA-seq. (**a**) The bar plots show the number and type of droplets that pass the indicated filtering method in the differentiating preadipocytes (DiffPA), mouse brain, and six human frozen adipose tissue (AT) snRNA-seq samples. The height of the blue bar indicates the number of nuclear droplets that pass filtering, while the height of the red bar indicates the number of background droplets. DIEM filtering tends to result in a higher number and proportion of nuclear droplets. Background and nuclear droplets are defined using the percent spliced reads. (**b**) The percent of reads spliced is shown in a boxplot for droplets that pass the indicated filtering method in the DiffPA, mouse brain, and six AT snRNA-seq samples. The horizontal red line indicates the sample-specific midpoint, where droplets above and below are background and nuclear, respectively. A Mann-Whitney U test was performed between DIEM and EmptyDrops^12^, and DIEM and quantile-filtered droplets. DIEM shows a decrease in percent spliced reads for all comparisons (black bar and asterisks) except for AT4 with EmptyDrops (red bar and asterisks). P-values were corrected for multiple testing using Bonferroni and are shown in the upper portion of the plot (*p < 0.05; **p < 0.005; ***p < 0.0005). (**c**) UMAP^33^ visualization of clusters after filtering with the indicated method in the combined adipose tissue snRNA-seq data set. Clusters were identified with Seurat^20^ and classified as adipocyte (Adp), doublet (Dblt), myeloid (Myl), T cell, mast, and stromal (Stm) cell types according to their up-regulated genes. A cluster was classified as debris (Dbr) if it had a mean percent of spliced reads above 50%.

We ran DIEM on the adipocyte, mouse brain, and six adipose tissue samples. We observed that DIEM removed droplets across a range of total UMI counts (Fig. 3c). We then evaluated the extent of extranuclear contamination, as well as its effect on clustering, across the quantile, EmptyDrops, and DIEM methods. We first quantified the number of nuclear and background droplets that passed filtering in each of the 8 experiments. In the DiffPA data set, the DIEM and quantile methods kept a larger proportion of nuclear droplets. Among the passing droplets, 1,337 of 1,339 (99.9%), 1,360 of 1,579 (86.1%), and 908 of 944 (96.2%) were nuclear in the DIEM, EmptyDrops, and quantile droplets, respectively (Fig. 4a). In the mouse brain data set, all three methods produced similar results. We found that 1,850 of 2,010 (92.0%), 1,868 of 2,080 (89.8%), and 1,832 of 2,083 (87.6%) passing droplets were nuclear in the DIEM, EmptyDrops, and quantile droplets, respectively (Fig. 4a). Across all 6 adipose tissue samples, 12,117 of 12,715 (95.7%), 10,110 of 11,502 (87.9%), and 9,578 of 11,331 (84.5%) passing droplets were nuclear in the DIEM, EmptyDrops, and quantile droplets, respectively (Fig. 4a). We further investigated these filtering methods in each of the adipose tissue samples. We found that the percent of DIEM passing droplets that were nuclear was significantly higher when compared to EmptyDrops and the quantile approach (paired Wilcoxon p = 0.03). Overall, the DIEM method tended to keep a higher number and proportion of nuclear droplets in the 8 snRNA-seq experiments.

Next, we compared the fraction of spliced reads in individual droplets for the independent experiments using a Mann–Whitney U test (Fig. 4b). When compared to EmptyDrops, DIEM had a significantly lower spliced reads fraction for the DiffPA, AT2, AT3, and AT6 samples. Although DIEM droplets had a lower mean of percent spliced reads for the AT4 sample, the Mann–Whitney U test yielded a significantly higher rank for the spliced reads fraction when compared to EmptyDrops (Bonferroni-corrected p < 0.05; Fig. 4b). When compared to the quantile-passing droplets, DIEM had a significantly lower spliced reads fraction for the mouse brain and 5 of the 6 adipose tissue samples (Bonferroni-corrected p < 0.05; Fig. 4b). None of the quantile-filtered droplets produced samples with a lower percent of reads spliced than DIEM, suggesting the quantile droplets contain more ambient RNA. Taken together, these results suggest that the DIEM-passing droplets comprise more nuclear droplets when using the percent of reads spliced as a measure of contamination.

We then looked at the effect of filtering on clustering results. We clustered passing droplets using Seurat^20^ to unbiasedly evaluate the clustering results based on each of the three methods. We considered clusters with a mean percent spliced reads of at least 50% as debris clusters and classified those with less than 50% as cell types consistent with expressed marker genes (see “Methods”). In the DiffPA dataset, DIEM removed a debris cluster that was present after filtering with both the quantile and EmptyDrops methods (Fig. S8a, b). Additionally, both EmptyDrops and DIEM identified a low count cluster that showed evidence of containing nuclei (Fig. S9), highlighting how a hard count threshold can result in removing cell types with lower counts. In the mouse brain data set, both EmptyDrops and DIEM removed a background cluster (median spliced reads 88.5%) that was present in the quantile method (Fig. S8c, d). For the adipose tissue data set, we combined the 6 individual filtered data sets and ran Seurat clustering with a resolution value of 2 to accommodate the larger number of droplets. DIEM resulted in 21 clusters, while EmptyDrops and the quantile method yielded 23 clusters. We then classified clusters based on their marker genes (Figs. 4c–e and S10). None of the DIEM clusters had a mean percent of reads spliced above 50% (Fig. S11a). However, EmptyDrops filtering resulted in 3 debris clusters while the quantile approach yielded 4 (Fig. S11a). There was a high overlap of over 50% in the major cell types between all 3 methods, but clusters consisting of smaller numbers of droplets tended to be spread across related cell types (Fig. S11b). Overall, these results suggest that DIEM preserves cell types while removing clusters characterized by high extranuclear contamination when compared to the EmptyDrops and quantile approaches.

### DIEM filtering removes a higher proportion of contaminated droplets and clusters in snRNA-seq

**Figure 5:**
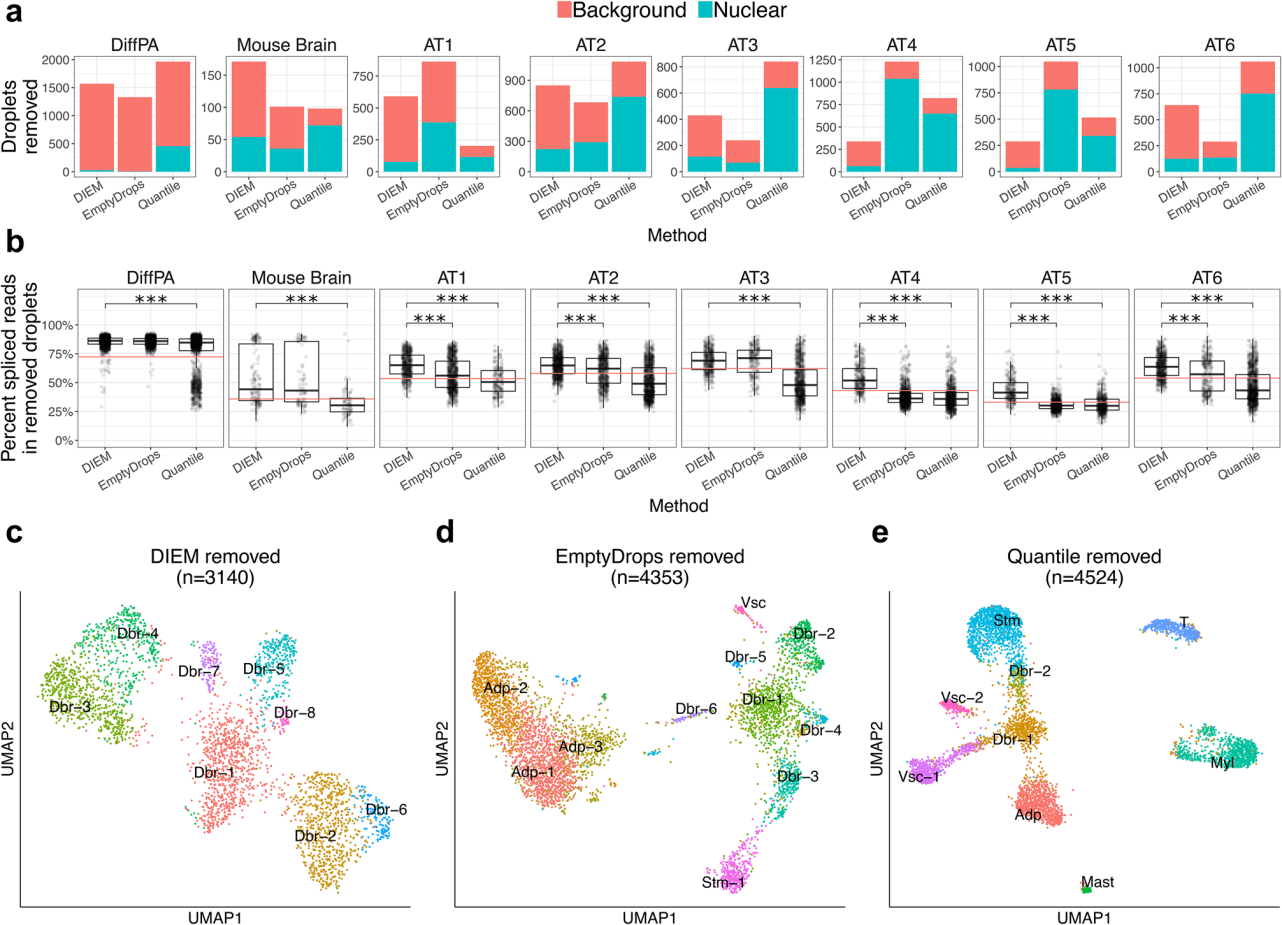
DIEM filtering removes fewer numbers of nuclei in snRNA-seq. (**a**) The bar plots show the number and type of droplets that are removed by the indicated filtering method in the differentiating preadipocytes (DiffPA), mouse brain, and six human frozen adipose tissue (AT) snRNA-seq samples. The height of the blue bar indicates the number of nuclear droplets that are removed while the height of the red bar indicates the number of background droplets. Background and nuclear droplets are defined using the percent spliced reads. DIEM filtering tends to result in a higher number and proportion of nuclear droplets. Removal of large numbers of nuclear droplets and low numbers of background droplets indicates poor performance. (**b**) The percent of reads spliced is shown in a boxplot for droplets removed by the filtering method in the DiffPA, mouse brain, and six AT snRNA-seq samples. The horizontal red line indicates the sample-specific midpoint, where droplets above and below are background and nuclear, respectively. A Mann-Whitney U test was performed between DIEM and EmptyDrops^12^, and DIEM and quantile removed droplets. DIEM shows an increase in percent of reads spliced for all comparisons. P-values were corrected for multiple testing using Bonferroni and are shown in the upper portion of the plot (*p < 0.05; **p < 0.005; ***p < 0.0005). (**c**) UMAP^33^ visualization of clustering of removed droplets with the indicated method in the combined adipose tissue snRNA-seq data set. Clusters were classified as adipocyte (Adp), doublet (Dblt), myeloid (Myl), T cell, mast, and stromal (Stm) cell types according to their up-regulated genes. A cluster was classified as debris (Dbr) if it had a mean percent of spliced reads above 50%.

We next investigated whether filtering incorrectly removed nucleus-containing droplets and possibly true cell types. Similar to the above analysis, we quantified the number of nuclear and debris droplets that were removed by each of the three methods. In the DiffPA dataset, 1,542 of 1,570 (98.2%), 1,325 of 1,330 (99.6%), and 1,508 of 1,965 (76.7%) removed droplets were background droplets after DIEM, EmptyDrops, and quantile filtering, respectively (Fig. 5a). The DIEM and EmptyDrops methods performed similarly in the mouse brain data set, and both outperformed the quantile approach. We found that 117 of 171 (68.4%), 65 of 101 (64.4%), and 26 of 98 (26.5%) removed droplets were classified as background in the DIEM, EmptyDrops, and quantile filtering, respectively (Fig. 5a). Across all adipose tissue samples, we found that the DIEM-removed droplets consisted of a higher proportion of background-derived droplets. We found that 2,499 of 3,140 (79.6%), 1,651 of 4,353 (37.9%), 1,290 of 4,524 (28.5%) removed droplets were background droplets in the DIEM, EmptyDrops, and quantile filtering, respectively (Fig. 5a). We investigated the percent of background droplets in those removed in each of the 6 adipose tissue samples as well. The percent of the removed droplets that were background was significantly higher with DIEM when compared to EmptyDrops and the quantile approach (paired Wilcoxon p = 0.03). We found that EmptyDrops incorrectly removed a much higher number of nuclear droplets in the AT4 and AT5 samples (Fig. 5a). EmptyDrops filtered out 1,038 in the AT4 and 782 in the AT5 samples, whereas DIEM removed 63 and 36 nuclear droplets, respectively (Fig. 5a). Overall, DIEM tended to remove a higher number and proportion of background droplets than EmptyDrops or the quantile approach.

We next investigated the amount of extranuclear contamination in the individual filtered-out droplets using a Mann–Whitney U test. We found that DIEM removed more background droplets with a significantly higher percent of reads spliced in all 8 experiments when compared to the quantile approach (Bonferroni-corrected p < 0.05; Fig. 5b). When compared to EmptyDrops, DIEM-removed droplets had a significantly higher percent of spliced reads for 5 of the 6 adipose tissue samples (Bonferroni-corrected p < 0.05; Fig. 5b). Neither the EmptyDrops nor the quantile method resulted in significantly more contamination in the removed debris droplets than DIEM. These results suggest that the DIEM-removed droplets contained fewer nuclei when using the percent of reads spliced as a measure of contamination.

Among the droplets removed by the three filtering methods, we sought further evidence that they originated from cell types. We clustered the removed droplets in the adipose tissue and looked to see if they consisted of biological cell types. We again considered clusters with a mean percent of reads spliced of at least 50% as debris clusters and classified those with less than 50% as cell types consistent with expressed marker genes (Figs. S1 and S12). Among the 8 clusters present in the DIEM-removed droplets, all had an average percent of reads spliced above 50%, suggesting that these consist of largely contaminated droplets (Figs. 5c and S12a). The EmptyDrops-removed droplets formed 11 clusters, 6 of which were debris. The other 5 clusters consisted of adipocyte, vascular, and stromal cell types (Figs. 5d and S12a). The quantile-removed droplets formed 7 cell type and 2 debris clusters. The cell type clusters consisted of adipocyte, stromal, T cell, myeloid, and mast cell types (Figs. 5e and S12a). Taken together, we found that the clusters formed by DIEM-removed droplets had more extranuclear contamination than those from EmptyDrops and the quantile method.

Interestingly, we found that the debris clusters formed by all filtering methods exhibited cell type-specific expression (Fig. S12b–e). This suggests that nucleus-containing droplets exhibit a range of extranuclear contamination in snRNA-seq experiments from frozen tissue. Furthermore, we found that droplets that had high read counts of the macrophage marker *CD14*^21^ tended to have higher extranuclear contamination and were more often filtered out by DIEM (Figs. S10f and S12d). This suggests that *CD14* + macrophages are more susceptible to damage or contamination and may imply that nuclei isolation or the snRNA-seq assay may introduce a bias in cell type capture.

### DIEM filtering removes debris from single-cell RNA-seq

**Figure 6:**
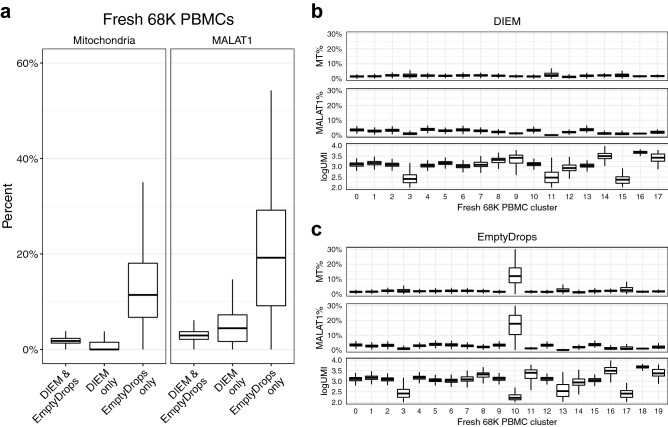
DIEM filtering in single-cell RNA-seq of fresh PBMCs results in robust cell type identification. (**a**) Boxplots showing the percent of unique molecular indices (UMIs) mapping to the mitochondria (left) and the percent of MALAT1 UMIs (right) in the fresh 68 K peripheral blood mononuclear cells (PBMC) data set^16^. The DIEM and EmptyDrops^12^ set includes the droplets identified by both DIEM and EmptyDrops (n = 75,658), while the EmptyDrops only set (n = 1,927) and the DIEM only set (n = 189) include droplets uniquely kept by each method. The droplets uniquely kept by EmptyDrops have a higher percent of reads aligned to the mitochondrial and MALAT1 genes, consistent with a ruptured cell membrane. (**b**, **c**) Boxplots show the percent of UMIs aligning to the mitochondrial genome (MT%), to the nuclear-localized MALAT1^18^ (MALAT1%), and the log total number of UMIs in a droplet for clusters in the PBMC single-cell RNA-seq data set. These measures are plotted for the (**b**) clusters from the DIEM-kept droplets and the (**c**) clusters from the EmptyDrops-kept droplets. Clusters were identified with Seurat^20^. The droplets uniquely kept by EmptyDrops form a distinct cluster with high MT% and MALAT1%.

In addition to filtering snRNA-seq, we also investigated whether our approach could be applied to single-cell RNA-seq data. We found that the debris scoring approach of individual droplets did not effectively distinguish empty vs. cell droplets in the 68,000 PBMC single-cell RNA-seq experiment^16^ (Fig. S13). DIEM gave a high debris score to a cell type cluster with high read counts, suggesting the debris-enriched genes and thus the debris score were less specific in discriminating debris droplets from all cell types in this PBMC single-cell RNA-seq data set. Although the threshold could be increased to accommodate this cell type, we found that simply removing droplets belonging to the fixed debris cluster was effective in removing empty droplets in the single cell RNA-seq data. Both DIEM and EmptyDrops kept all 69,981 droplets that had at least 200 genes detected. To evaluate the effect of filtering, we removed this threshold to more completely characterize the two methods. Among 78,024 comparable droplets with at least 100 UMI counts, EmptyDrops kept 77,585 and DIEM kept 75,847. The 1,927 droplets unique to EmptyDrops showed a high percent of reads aligning to the mitochondria and to *MALAT1* (Fig. 6a). The 189 unique droplets to DIEM showed MT% and MALAT1% levels similar to the shared droplets that passed both filtering methods (Fig. 6a). Although these metrics no longer serve as negative and positive controls as they do in snRNA-seq, they are consistent with a ruptured cell membrane. This suggests that EmptyDrops retains droplets with dying cells whereas DIEM removes them. We next evaluated the clusters formed by these droplets. DIEM-filtered droplets formed 18 clusters while EmptyDrops resulted in 19 clusters. As expected, there was a general one-to-one correspondence between the clusters. However, the droplets with high MT% and MALAT1% formed a cluster that was absent in the DIEM results (Fig. 6b, c). Overall, we found that EmptyDrops and DIEM provided similar results in the PBMC single-cell RNA-seq data.

## Discussion

The snRNA-seq approach is an adaptation of scRNA-seq that allows for cell-type identification when isolation of a cell suspension is not possible, such as in frozen tissues. We have shown here that snRNA-seq is subject to background contamination from extranuclear RNA and that it can drive spurious clusters and false positive cell-types if not properly accounted for. We also show that current methods, such as the commonly applied hard count threshold, do not effectively address this problem in snRNA-seq. To this end, we searched for and found differences in the gene expression profiles from the debris and cell types. This motivated us to develop DIEM in order to use the RNA profile of a droplet for filtering contaminated snRNA-seq experiments. We found that DIEM efficiently removed debris-contaminated droplets while preserving cell types in snRNA-seq data from fresh cells, fresh tissue, and frozen tissue inputs.

DIEM first clusters all droplets generated from the experiment to identify droplets belonging either to the debris cluster or putative cell type clusters. This allows for a general separation of droplets into debris and cell type groups, and thus more accurate differential expression analysis between background and cell type droplets. Although it is possible to simply remove droplets that cluster as debris, we found that clusters with high amounts of contamination still existed in snRNA-seq. Therefore, scoring and filtering individual droplets allow for finer classification as well as quantification of the amount of contamination. The debris score threshold that removes droplets can be adjusted according to the desired tolerance for contamination. In addition, this estimate can be used as a covariate in downstream analyses, such as clustering and differential expression analyses. The debris score, however, cannot be assumed to exist on the same scale in independent experiments. The scores are normalized relative to the best and worst cluster means within the sample. Thus, when integrating multiple samples, the debris scores may not be comparable, particularly if the distribution of extranuclear RNA is different across the samples. Finally, the scoring approach relies on accurate estimation of debris-enriched genes. If there are little or no genes that are increased in the background distribution, the resulting debris score will likely be inaccurate. Although we found that the debris scores in all 8 snRNA-seq experiments were correlated with the level of extranuclear contamination as measured by the percent of reads spliced, this was less successful in the PBMC single-cell RNA-seq data. This may be due to smaller differences between the background and cell type profiles in scRNA-seq.

Since we found that extranuclear RNA contamination exists across a wide range of UMI counts, using a hard count threshold underperformed in comparison to EmptyDrops and DIEM. Both DIEM and EmptyDrops^12^ remove droplets based on their expression distribution. When compared to the EmptyDrops method^12^, however, we found that DIEM had a higher accuracy in filtering heterogeneous snRNA-seq data sets as assessed by the percent of reads that are spliced as a metric of extranuclear RNA. EmptyDrops, however, was originally developed and tested on single cell data and thus, the assumptions behind the model are different than that of DIEM. EmptyDrops only models the background RNA distribution and uses Monte Carlo sampling to determine how significant the deviation of a droplet is from it. It also safeguards from removing cell-types that are similar to the background by assuming that all droplets above a calculated knee point are true cell-containing droplets. DIEM directly models cell types by clustering and this may allow for more accurate grouping of debris droplets. We have shown that the difference between the cell types and the debris are within the same order of magnitude as the differences between the cell types, highlighting the need to account for heterogeneity. We found that both EmptyDrops and the quantile approach removed more nuclear droplets and kept a higher proportion of contaminated droplets, but the major adipose cell types were still identified. However, since the DIEM-filtered droplets contained less extranuclear contamination, the resulting clusters were also characterized by less debris on average. This is beneficial for both accurate cell type clustering and identification.

Even though snRNA-seq recovers less RNA than scRNA-seq and thus retrieves less information about cell types, there are advantages to using nuclei over cells. For example, snRNA-seq has been shown to reduce dissociation biases present in scRNA-seq, leading to more accurate profiling of cell types in tissue^22^. Another important reason to use snRNA-seq is that scRNA-seq may be practically impossible. This can occur with frozen tissues, since thawing cells is known to lyse the outer membranes and preclude a suspension of single cells required for droplet-based technologies^3^. This prevents the application of scRNA-seq to biobanked snap-frozen human tissues. In order to leverage existing, phenotyped human datasets with biobanked tissues, snRNA-seq may be the only viable option to profile cell types. We have shown that snRNA-seq of frozen tissue results in contamination of droplets across a large range of UMI counts, making it difficult to remove background debris while maintaining an accurate cell type composition of the tissue. Even from fresh tissue and cells, we still observed downstream clusters affected by the extranuclear RNA. Therefore, we expect DIEM to help produce cleaner snRNA-seq data sets from a variety of input sources, but especially from frozen tissues.

We focused the application of our approach on snRNA-seq data because there is a pressing need for debris filtering in data sets with lower RNA content. In single-cell RNA-seq, the higher RNA content of cells typically allows the total UMI count of a droplet to serve as a sufficient discriminator between debris and cells^3^, although this may not always be the case^12^. However, running scRNA-seq on fresh human tissue at a large scale may be prohibitively difficult considering the requirement to immediately process a fresh biopsy for scRNA-seq. Therefore, snRNA-seq of frozen tissues offers a viable alternative to process samples at a higher throughput. Our method was designed to computationally remove background debris contamination from snRNA-seq data of frozen tissues. We expect that DIEM will enable the analysis of a larger number of samples from frozen tissue snRNA-seq data, thereby removing the need to coordinate the acquisition of fresh tissue samples and processing of single cell libraries.

## Methods

### Single-nucleus RNA-seq of human subcutaneous adipose tissue, differentiating preadipocytes, and mouse brain

Frozen subcutaneous adipose tissue was processed separately for each of the 6 samples. Tissue was minced over dry ice and transferred into ice-cold lysis buffer consisting of 0.1% IGEPAL, 10 mM Tris–Hcl, 10 mM NaCl, and 3 mM MgCl_2_. After a 10 min incubation period, the lysate was gently homogenized using a dounce homogenizer and filtered through a 70 μm MACS smart strainer (Miltenyi Biotec #130-098-462) to remove debris. Nuclei were centrifuged at 500×*g* for 5 min at 4 °C and washed in 1 ml of resuspension buffer (RSB) consisting of 1X PBS, 1.0% BSA, and 0.2 U/μl RNase inhibitor. We further filtered nuclei using a 40 μm Flowmi cell strainer (Sigma Aldrich # BAH136800040) and centrifuged at 500×*g* for 5 min at 4 °C. Pelleted nuclei were re-suspended in wash buffer and immediately processed with the 10X Chromium platform following the Single Cell 3′ v2 protocol. After library generation with the 10X platform, libraries were sequenced on an Illumina NovaSeq S2 at a sequencing depth of 50,000 reads per cell. Reads were aligned to the GRCh38 human genome reference with Gencode v26 gene annotations^23^ using the 10X CellRanger 2.1.1 pipeline. A custom pre-mRNA reference was generated to account for unspliced mRNA by merging all introns and exons of a gene into a single meta-exon.

We obtained and cultured the primary human white preadipocyte cells as recommended by PromoCell (PromoCell C-12731, lot 395Z024) for preadipocyte growth and differentiation into adipocytes. Cell media (PromoCell) was supplemented with 1% penicillin–streptomycin. We maintained the cells at 37 °C in a humidified atmosphere at 5% CO2. On day 6 of differentiation, we rinsed the cells with 1 × PBS and added ice-cold lysis buffer (3 mM MgCl_2_, 10 mM Tris–HCl, 0.5% Igepal CA-630, 10 mM NaCl). The cells were gently scraped from the plate and centrifuged at 500×*g* for 5 min at 4 °C. Nuclei were washed with 1 ml of resuspension buffer (RSB; 1% BSA, 100 μl RNase inhibitor in 1 × PBS) and centrifuged again to remove cellular debris. After the second centrifugation, nuclei were washed with 1 ml RSB and filtered through a 40 μm filter. Cells were counted, then centrifuged again and resuspended in the proper volume of RSB to obtain 2000 nuclei/μl. The 10X library preparation, sequencing, and data processing were done using the same protocol as for the adipose tissue.

For the mouse brain data, we downloaded the raw UMI count data matrix from the 10X website. The data set titled “2K Brain Nuclei from an Adult Mouse (> 8 weeks)” was downloaded from https://support.10xgenomics.com/single-cell-gene-expression/datasets/2.1.0/nuclei_2k. The 10X human 68K PBMC data were downloaded from https://support.10xgenomics.com/single-cell-gene-expression/datasets/1.1.0/fresh_68k_pbmc_donor_a.

### Filtering droplets using a quantile threshold, EmptyDrops, and DIEM

Common methods for removing debris from snRNA-seq data rely on using a hard count threshold^3,8–11^. In the three data sets, we applied a quantile-based cutoff, similar to that implemented by the 10X CellRanger software. Droplets are ranked in decreasing order of total counts. The 99th percent quantile of the top *C* barcodes of total counts is divided by 10 to obtain the threshold *T*, where *C* is 3,000 for our analyses^16^. The 99th percentile is used to exclude any doublets from the derivation. Droplets with greater than or equal to *T* counts were included as nuclei. For comparison with EmptyDrops^12^, we ran the method using default parameters. EmptyDrops calculates a Monte Carlo p-value that gives the probability that a droplet’s expression profile is the same as that of the ambient RNA. We removed droplets with a false discovery rate (FDR) q value greater than 0.05. For the six adipose tissue samples, we applied filtering to each sample independently, as these are the result of individual experiments. We also tested DIEM filtering after combining the counts in the 6 adipose tissue samples and observed similar results (Fig. S14).

### Estimating extranuclear RNA contamination in droplets from snRNA-seq

We used three metrics to estimate contamination of background RNA in the snRNA-seq data sets. We quantified the fraction of spliced reads using velocyto^17^. The BAM files from CellRanger were sorted by barcode ID using samtools^24^, and spliced, unspliced, and ambiguous read counts were quantified for each gene. We then removed mitochondria (MT) reads to avoid confounding of the estimates, as the MT genes do not have introns. For each droplet, the unspliced and spliced UMI counts were added, and the percent of reads spliced was calculated as the fraction of all spliced reads over the sum of spliced and unspliced reads. We calculated the percent of UMIs aligned to the mitochondria (MT%) as the sum of reads aligned to the mitochondrial genome over the droplet’s total UMI counts. The percent of UMIs aligned to *MALAT1* (MALAT1%) was calculated similarly.

We classified droplets as background or nuclear according to their percent of reads spliced. Since this metric showed a bimodal distribution that was distinct in each sample, we calculated the midpoint between the two distributions. To do so, we modeled the percent of reads spliced as a mixture of two gaussians. We fit the parameters using EM with the R package mixtools^25^. Then, the midpoint was calculated as the value in which the probability density was equal in the two distributions. Droplets with a percent of reads spliced above and below the midpoint were classified as background and nuclear, respectively. For clusters, we specified those with an average percent of reads spliced of at least 50% as debris and classified those with less than 50% as nuclear, as we observed that 50% was the average value of the midpoint across the experiments and that the 6 adipose tissue samples were combined.

### Differential expression between nuclear-enriched and debris-enriched droplets

To identify genes differentially expressed (DE) between the background-enriched and nuclear-enriched groups, we set a hard count threshold to naively assign droplets to either group. Droplets with total UMI counts below 100 and greater than or equal to 100 were assigned to the background-enriched and nuclear-enriched groups, respectively. This ensures that the majority of droplets containing nuclei are found in the nuclear-enriched group. For each gene, reads were summed across all droplets in each of the two groups to estimate the RNA profiles. Read counts were normalized using trimmed mean of M-values (TMM) as implemented in edgeR^26,27^. For identifying differentially expressed genes, we used a paired design with the six adipose tissue samples by treating the background-enriched and nuclear-enriched counts of an individual as a paired sample (total n = 12). We then used the edgeR package^26,27^ to run differential expression. We only kept genes with a counts per million (CPM) of greater than 0 in at least 6 of the 12 groups. Next, we used the estimateDisp function to estimate the dispersion with the paired design matrix. The quasi-likelihood fit and F test functions glmQLFit and glmQLFTest were used to calculate statistical significance. We adjusted for multiple testing using a Bonferroni-corrected p-value threshold of 0.05.

To identify DE genes between the debris and cell types, we used the clusters identified after quantile-based filtering to approximate the cell types. For each of the six samples, we subsampled the debris droplets (with total UMI counts less than 100) to 9,000 droplets to obtain a similar read depth as contained in the cell type groups. For the debris and cell type groups, reads were summed across the corresponding droplets to obtain the RNA profile used as input. Differential expression was performed by comparing debris vs. cell type or cell type vs. all other cell types using a paired design. The filtering and analysis was performed in the same manner as the debris vs. nuclear DE analysis above.

### DIEM algorithm

DIEM first assigns droplets as originating from debris or cell types, and then calculates the level of contamination within droplets using the debris-enriched genes. To assign droplets to either debris or cell types, our filtering approach models droplet-based single-cell or single-nucleus data with a mixture of multinomial distributions. Particularly, droplet read counts are assumed to follow a multinomial with parameters conditional on the cluster. However, the parameters and droplet assignments are unknown for the droplets of interest. In addition, it is assumed that the majority of low count droplets contain ambient RNA. Therefore, we estimate the parameters of the model using semi-supervised expectation maximization (EM)^13,14^. This allows us to calculate the probability of the latent group variable given the data, and thus group debris and cell type droplets. To initialize the parameters for EM, we cluster the droplets with k-means, where the number of cell types k is specified by the user. We include only droplets with at least 200 genes detected in this initialization step to avoid fitting clusters driven by empty debris droplets. After fitting the mixture model with EM, we calculate a debris score for each droplet based on the expression of genes enriched in the debris set.

In more detail, let *X* denote a *g* x *N* matrix containing the read/UMI counts from a single-cell or single-nucleus data set with *g* genes and *N* droplets. We include droplets with at least 1 read/UMI count. Our goal is to assign the *N* droplets into one of *K* + 1 groups (*K* cell types and debris). We define *x*_*i*_ as the *i*th column of *X* giving the counts of droplet *i* and assume that it follows a multinomial distribution with the gene probabilities ${\boldsymbol{\alpha }}_{{\varvec{k}}}$ = *p*_*1,k*_, …, *p*_*G,k*_ conditional on group $k\in \{1,...,K, K+1\}$. We model droplet expression using a multinomial distribution, and further model cell types and debris using a mixture of multinomials. The log-likelihood of the data is therefore:

$$logP({\varvec{X}}) ={\sum }_{i=1}^{N}log\left({\sum }_{k=1}^{K+1}{\pi }_{k}Mult({{\varvec{x}}}_{{\varvec{i}}}|{\boldsymbol{\alpha }}_{{\varvec{k}}},{u}_{i})\right)$$

Here, *u*_*i*_ is the total number of read/UMI counts in droplet *i*, ${\boldsymbol{\alpha }}_{{\varvec{k}}}$ contains the multinomial parameters for group *k*, *π*_*k*_ is the mixing coefficient for group *k*, and *Mult* denotes the probability mass function of the multinomial distribution. Since an analytical solution cannot be derived based on the likelihood of the model, we define ${z}_{i}\in \{1,...,K\}$ for each *i* as a latent indicator variable that describes the cell type or debris origin of the droplet. The complete log-likelihood of the data and the latent variables $Z={\left\{{z}_{i }\right\}}_{i=1}^{N}$ now becomes:

$$logP({\varvec{X}},{\varvec{Z}}) ={\sum }_{i=1}^{N}{\sum }_{k=1}^{K+1}{\mathbb{I}}\left\{{\mathrm{z}}_{\mathrm{i}}=\mathrm{k}\right\}[\mathrm{log}{\pi }_{k}+\mathrm{ log}Mult({{\varvec{x}}}_{{\varvec{i}}}|{\boldsymbol{\alpha }}_{{\varvec{k}}},{u}_{i})]$$

where ${\mathbb{I}}\left\{{\mathrm{z}}_{\mathrm{i}}=\mathrm{k}\right\}$ is an indicator variable for the assignment of droplet i. This formulation allows us to employ an EM algorithm and estimate the parameters ${\alpha }_{1}$, …, ${\alpha }_{k}$ and *π*_*1*_, …, *π*_*k*_ by maximizing the expected complete data log-likelihood. The latent indicator variables for the debris droplets with UMI counts below 100 remain fixed, thus effectively resulting in a semi-supervised EM.

Although it is possible to remove droplets that contain a high posterior probability of belonging to the fixed debris cluster, we employ a scoring strategy to quantify the level of contamination within individual droplets. This provides both a finer resolution in debris filtering and a direct estimate that can be used as a covariate in downstream analysis.

Droplets are divided into a test set and a debris set. The test set consists of the droplets we would like to classify, while the debris set consists of droplets that we assume to contain debris with high probability. The labels of droplets in the debris set are fixed throughout the EM iterations, while those of the test set are allowed to change. We define the test set as those droplets with at least *T* total counts, where we set *T* to a default value of 100. Only expressed genes with a counts per million (CPM) > 0 are included in the analysis.

### Initialization of parameters for EM

The EM algorithm requires starting values for the parameters of the model. The parameters $\boldsymbol{\alpha }$ and ***π*** are initialized from the PCs of the cluster set of droplets using k-means. A proper initialization is important because mixture models can be sensitive to local optima^28^. Therefore, we run k-means, which has been shown to provide reasonable initial values for EM^29,30^. As the test set may contain many more empty droplets than the droplets of interest, we further define a cluster set for k-means as those droplets with at least 200 genes detected ^20^. K-means is run on the on the first 30 PCs of the data using the kmeans function in R. Before running PCA, we first select the top *V* = 2,000 variable genes^20,31^. To do so, we first account for the relationship between the mean and variance^31,32^. The mean and variance of the raw gene counts are calculated and log transformed. To learn the relationship, we fit a locally weighted smoothing (LOESS) regression line between the normalized mean and variance using the loess function in R with a span = 0.3. We correct the variance for the expression level of a gene by subtracting the fitted variance from the observed variance. Finally, we rank the genes by their standardized variance and take the top *V* = 2,000 genes. PCA is run on the normalized counts, where the total droplet read counts are scaled to sum to the median read depth and then log transformed. PCA is performed on the adjusted counts on the cluster set and the variable genes, and the top 30 principal components are returned. Finally, k-means is run on these PCs, with the number of cell types k specified by the user. We use k = 20 for all experiments in the manuscript, unless otherwise specified. The initial parameters are estimated from the droplets assigned to these resulting clusters.

### Estimation

The EM algorithm iteratively estimates the parameters and the posterior probabilities. Given $\widehat{\boldsymbol{\alpha }}$ and $\widehat{{\varvec{\pi}}}$, estimates of $\boldsymbol{\alpha }$ and ***π***, we calculate the posterior probability that droplet *X*_*i*_ belongs to cluster *k*

$$p\left({z}_{i}=k \right|{\boldsymbol{ }{\varvec{x}}}_{{\varvec{i}}},{\widehat{\boldsymbol{\alpha }}}_{{\varvec{k}}},\widehat{{\varvec{\pi}}})=\frac{p\left({\widehat{{\varvec{\pi}}}}_{k}\right)p\left({{\varvec{x}}}_{{\varvec{i}}} \right| {{z}_{i}=k , \widehat{\boldsymbol{\alpha }}}_{{\varvec{k}}})}{{\sum }_{j=1}^{K}p\left({\widehat{{\varvec{\pi}}}}_{j}\right)p\left({{\varvec{x}}}_{{\varvec{i}}} \right| {{z}_{i}=j,\widehat{\boldsymbol{\alpha }}}_{{\varvec{j}}})}$$

where $p\left({X}_{i} \right| {{Z}_{i} , \alpha }_{k})$ follows the multinomial given the parameters ${\boldsymbol{\alpha }}_{{\varvec{k}}}$ for cluster *k* and $p\left({\pi }_{k}\right)$ follows a categorical distribution. The debris droplets with total counts below *T* = 100 have their *z*_*i*_ values kept fixed to the debris group. The maximum likelihood estimate of ${\boldsymbol{\alpha }}_{{\varvec{k}}}$ is calculated as the mean of the droplet counts weighted by their posterior probability of belonging to cluster *k*. We add a pseudocount of 10^−10^ to avoid collapsing the likelihood to 0. For *π*_*k*_, the maximum likelihood estimate is calculated as the sum of $p\left({\varvec{Z}}=k \right| {\varvec{X}})$ divided by the total number of droplets, so that *π*_*1*_, …, *π*_*K*_ sum to one. These two steps iterate during EM, and the algorithm converges when the change in parameters is below ε, which we set to 10^−4^. Droplets are assigned to the cluster that gives the maximum posterior probability.

### Debris scoring and filtering of individual droplets

We assign a debris score to individual droplets to obtain a finer estimate of the amount of contamination. After clustering, we specify the set of debris clusters as the fixed cluster as well as any clusters that have an average number of genes detected less than *d*, where we set *d* to 200. The cell type group then consists of all other clusters. We estimate the debris score by summing a droplet’s expression values of genes enriched in this debris set. To identify debris-enriched genes, we first run a Welch’s t-test between the test set droplets in the debris and cell type clusters. Read counts are normalized by scaling the counts to sum to 1 and then log normalizing after adding a constant of 1. Then, genes with a log fold change greater than 0 and an FDR-corrected p-value less than 0.05 are specified as debris-enriched genes.

The debris score is estimated by summing the normalized expression values of the debris-enriched genes. Since the magnitude of this score is dependent on the number and expression of the debris-enriched genes, we scale the scores. We calculate the mean of all clusters, subtract the scores by the lowest cluster average, and divide them by average of the droplets in the debris cluster(s). This has the effect of setting the average of the lowest cluster to 0 and the debris cluster(s) to 1, so that scores are scaled relative to these clusters. Droplets in the snRNA-seq experiments are filtered using a threshold for the debris score. We keep droplets with a normalized debris score below *t*, where we set *t* to 0.5, although we note that these can be adjusted by the user accordingly.

### Identifying cell types after filtering droplets

For all experiments, we ran a standardized clustering pipeline using Seurat v3.1.2^20^. After applying filtering, we only considered droplets with at least 200 genes detected ^4^ to ensure that each droplet had enough information for clustering. The count data were log-normalized using the NormalizeData function in Seurat, using a scaling factor equal to the median of total counts across droplets. For the six adipose tissue samples, we used a scaling factor equal to 1,000 to ensure that all samples were normalized equally. Additionally, we merged the normalized data of the six adipose tissue samples without batch correction, as we saw high overlap of clusters among the six samples (data not shown). The top 2,000 variable genes were then calculated using the FindVariableFeatures function.

Normalized read counts for each gene were scaled to mean 0 and variance 1. We calculated the first 30 PCs to use as input for clustering. We then ran the Seurat functions FindNeighbors and FindClusters with 30 PCs. In the FindClusters function, we used the default parameters with standard Louvain clustering and a default clustering resolution of 0.8, unless otherwise stated. For visualization, we ran UMAP^33^ on the 30 PCs with default values. To identify marker genes for each cluster, we ran a Wilcoxon rank-sum test using the function FindAllMarkers with default parameters and only.pos = TRUE. We corrected for multiple testing using a false discovery rate (FDR) threshold of 0.05. Clusters were classified as doublets if the top marker genes consisted of an identifiable mixture of top markers between two cell types.

### Ethics approval and consent to participate

All research was performed in accordance with the relevant institutional guidelines and regulations. Each of the 6 participants gave a written informed consent. The study protocol was approved by the Ethics Committee at the Helsinki University Hospital, Helsinki, Finland.

## Supplementary information

Supplementary file1 (PDF 5748 kb)

## Data Availability

The human single nucleus RNA-seq datasets generated and analyzed during the current study are available upon request from the corresponding author. The DIEM program is freely available for use at https://github.com/marcalva/diem. The code for the analysis is available at https://github.com/marcalva/DIEM2019.

## References

[1] Patel AP (2014). Single-cell RNA-seq highlights intratumoral heterogeneity in primary glioblastoma. Science.

[2] Baron M (2016). A single-cell transcriptomic map of the human and mouse pancreas reveals inter- and intra-cell population structure. Cell Syst..

[3] Macosko EZ (2015). Highly parallel genome-wide expression profiling of individual cells using nanoliter droplets. Cell.

[4] Habib N (2017). Massively parallel single-nucleus RNA-seq with DroNc-seq. Nat. Methods.

[5] Habib N (2016). Div-Seq: single-nucleus RNA-seq reveals dynamics of rare adult newborn neurons. Science.

[6] Krishnaswami SR (2016). Using single nuclei for RNA-seq to capture the transcriptome of postmortem neurons. Nat. Protoc..

[7] Nguyen QH, Pervolarakis N, Nee K, Kessenbrock K (2018). Experimental considerations for single-cell RNA sequencing approaches. Front. Cell Dev. Biol..

[8] Hu P (2017). Dissecting cell-type composition and activity-dependent transcriptional state in mammalian brains by massively parallel single-nucleus RNA-Seq. Mol. Cell.

[9] Lacar B (2016). Nuclear RNA-seq of single neurons reveals molecular signatures of activation. Nat. Commun..

[10] Lake BB (2016). Neuronal subtypes and diversity revealed by single-nucleus RNA sequencing of the human brain. Science.

[11] Zeng W (2016). Single-nucleus RNA-seq of differentiating human myoblasts reveals the extent of fate heterogeneity. Nucleic Acids Res..

[12] Lun ATL (2019). EmptyDrops: distinguishing cells from empty droplets in droplet-based single-cell RNA sequencing data. Genome Biol..

[13] Dempster APP, Laird NM, Rubin DB, Rubin DB (1977). Maximum likelihood from incomplete data via the EM algorithm. J. R. Stat. Soc..

[14] Do CB, Batzoglou S (2008). What is the expectation maximization algorithm?. Nat. Biotechnol..

[15] Nigam K, Mccallum AK, Thrun S, Mitchell T (2000). Text classification from labeled and unlabeled documents using EM. Mach. Learn..

[16] Zheng GXY (2017). Massively parallel digital transcriptional profiling of single cells. Nat. Commun..

[17] La Manno G (2018). RNA velocity of single cells. Nature.

[18] Miyagawa R (2012). Identification of cis- and trans-acting factors involved in the localization of MALAT-1 noncoding RNA to nuclear speckles. RNA.

[19] Hardison RC (2012). Evolution of hemoglobin and its genes. Cold Spring Harbor Perspect. Med..

[20] Stuart T (2019). Comprehensive integration of single-cell data. Cell.

[21] Ziegler-Heitbrock HWL, Ulevitch RJ (1993). CD14: cell surface receptor and differentiation marker. Immunol. Today.

[22] Wu H, Kirita Y, Donnelly EL, Humphreys BD (2019). Advantages of single-nucleus over single-cell RNA sequencing of adult kidney: rare cell types and novel cell states revealed in fibrosis. J. Am. Soc. Nephrol..

[23] Frankish A (2019). GENCODE reference annotation for the human and mouse genomes. Nucleic Acids Res..

[24] Li H (2009). The sequence alignment/map format and SAMtools. Bioinformatics.

[25] Benaglia T, Chauveau D, Hunter DR, Young DS (2009). Mixtools: an R package for analyzing finite mixture models. J. Stat. Softw..

[26] McCarthy DJ, Chen Y, Smyth GK (2012). Differential expression analysis of multifactor RNA-Seq experiments with respect to biological variation. Nucleic Acids Res..

[27] Robinson MD, McCarthy DJ, Smyth GK (2010). edgeR: a Bioconductor package for differential expression analysis of digital gene expression data. Bioinformatics.

[28] Biernacki C, Celeux G, Govaert G (2003). Choosing starting values for the EM algorithm for getting the highest likelihood in multivariate Gaussian mixture models. Comput. Stat. Data Anal..

[29] Steinley D, Brusco MJ (2011). Evaluating mixture modeling for clustering: recommendations and cautions. Psychol. Methods.

[30] McLachlan GJ, Lee SX, Rathnayake SI (2019). Finite mixture models. Annu. Rev. Stat. Appl..

[31] Brennecke P (2013). Accounting for technical noise in single-cell RNA-seq experiments. Nat. Methods.

[32] Hafemeister C, Satija R (2019). Normalization and variance stabilization of single-cell RNA-seq data using regularized negative binomial regression. Genome Biol..

[33] Becht E (2019). Dimensionality reduction for visualizing single-cell data using UMAP. Nat. Biotechnol..

