# Supplementary material for: Enhancing droplet-based single-nucleus RNA-seq resolution using the semi-supervised machine learning classifier DIEM
Source: Sci Rep. 2020 Jul 3;10:11019. doi: 10.1038/s41598-020-67513-5 (PMC7335186; doi:10.1038/s41598-020-67513-5)
Supplement: Supplementary file 1 — Supplementary file1 (PDF 5748 kb) [file 41598_2020_67513_MOESM1_ESM.pdf]

**Supplementary Information**

# Enhancing droplet-based single-nucleus RNA-seq resolution using the semi-supervised machine learning classifier DIEM


Marcus Alvarez[1*] malvarez@mednet.ucla.edu

Elior Rahmani[2*] elior.rahmani@gmail.com

Brandon Jew[3] brandon.jew@ucla.edu

Kristina M. Garske[1] kmgarske@g.ucla.edu

Zong Miao[1,3] zmiao@ucla.edu

Jihane N. Benhammou[1,5] jbenhammou@mednet.ucla.edu

Chun Jimmie Ye[4] jimmie.ye@ucsf.edu

Joseph R. Pisegna[1,5] jpisegna@mednet.ucla.edu

Kirsi H. Pietiläinen[6,7] kirsi.pietilainen@helsinki.fi

Eran Halperin[1,2,3] eranhalperin@gmail.com

Päivi Pajukanta[1,3,8**] ppajukanta@mednet.ucla.edu

1. Department of Human Genetics, David Geffen School of Medicine at UCLA, Los Angeles, CA, USA

2. Computer Science Department in the School of Engineering, UCLA, Los Angeles, CA, USA

3. Bioinformatics Interdepartmental Program, UCLA, Los Angeles, CA, USA

4. Institute for Human Genetics, Department of Epidemiology and Biostatistics, Department of Bioengineering and Therapeutic Sciences, UCSF, San Francisco, USA

5. Vache and Tamar Manoukian Division of Digestive Diseases, UCLA, Los Angeles, CA, USA

6. Obesity Research Unit, Research Programs Unit, Diabetes and Obesity, University of Helsinki, Biomedicum Helsinki, Helsinki, Finland

7. Obesity Center, Endocrinology, Abdominal Center, Helsinki University Central Hospital and University of Helsinki, Helsinki, Finland

8. Institute for Precision Health, David Geffen School of Medicine at UCLA, Los Angeles, CA, USA

*Equal first authorship

**Corresponding author:

Päivi Pajukanta, MD, PhD

Professor

Vice Chair, Department of Human Genetics

Director of Cardiometabolic Genomics, Institute for Precision Health

David Geffen School of Medicine at UCLA

Gonda Center, Room 6335B

695 Charles E. Young Drive South

Los Angeles, California 90095-7088, USA

Email: ppajukanta@mednet.ucla.edu

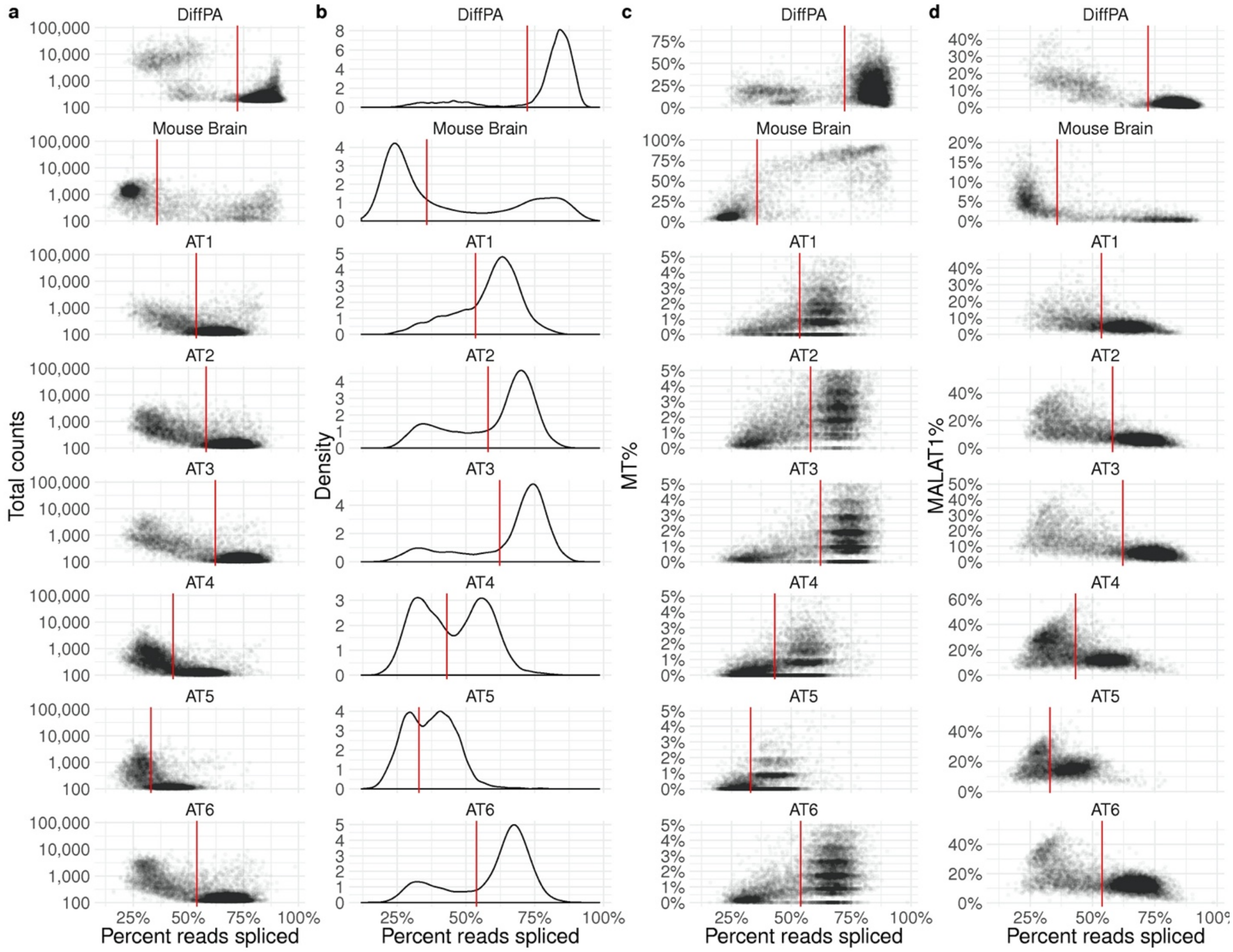


**Figure S1. The percent of reads spliced separates nuclear from background RNA in snRNA-seq droplets.**

The percent of reads spliced per droplet was calculated for each of the 8 independent samples in order to quantify extranuclear RNA contamination. To assess the effectiveness of this metric, we plotted the percent reads spliced against **(a)** total counts, **(b)** the density, **(c)** the percent of reads aligning to the mitochondria (MT%), and **(d)** the percent of reads aligning to *MALAT1* (MALAT1%). The human adipose tissue (AT) dataset was performed over 6 independent experiments. The spliced reads percent was calculated using Velocyto[17] after removing mitochondrial reads. As each sample demonstrated a distinct distribution of spliced reads, we estimated a cutoff (see methods) for each sample (vertical red line). Droplets with a percent of

reads spliced below the cutoff were classified as nuclear, and those greater than or equal to the cutoff as background.

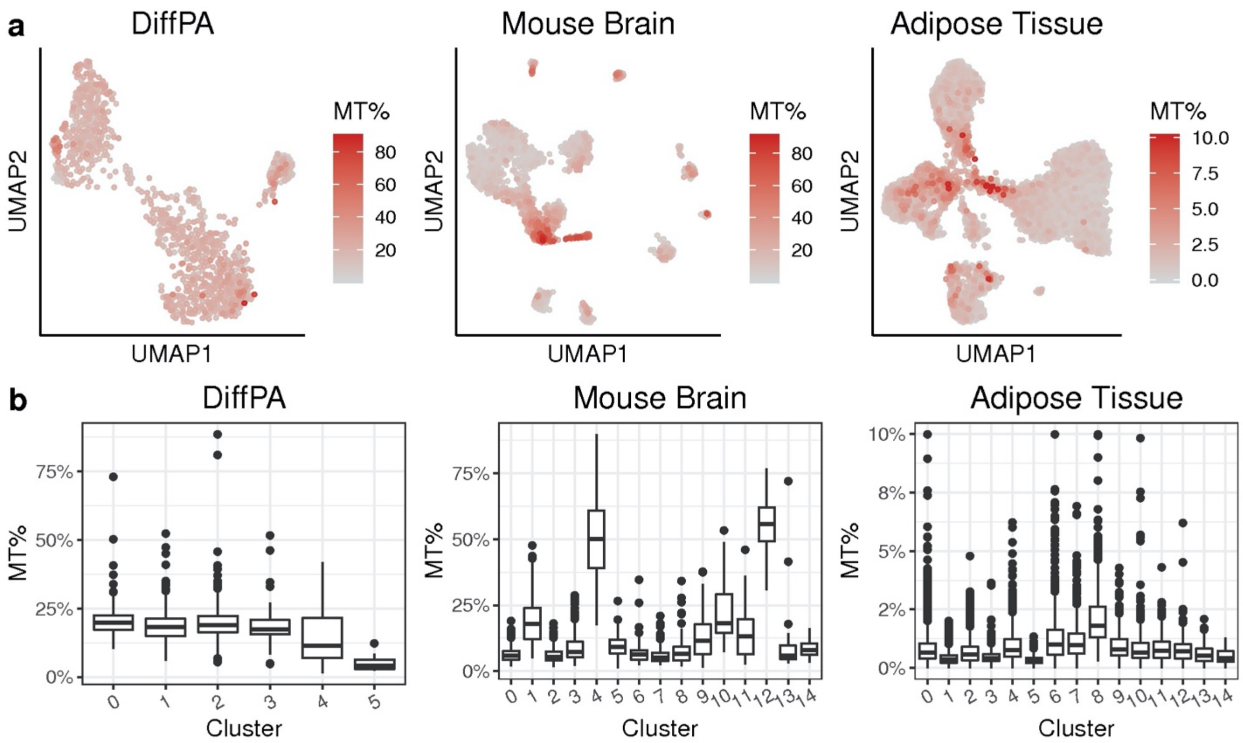


**Figure S2. A hard count threshold fails to remove contaminated droplets and results in spurious clusters when assessed using MT%.**

The estimation of background RNA when estimated using mitochondrial percent (MT%) in a droplet shows how a hard count threshold fails to remove contaminated droplets **a,** UMAP[33] visualizations for the differentiating preadipocytes (DiffPA), mouse brain, and human frozen adipose tissue (AT) data sets show clustering of contaminated droplets. **b,** boxplots of MT% in clusters after processing the filtered droplets with Seurat[20]. The quantile-based approach was used to select the hard count threshold.

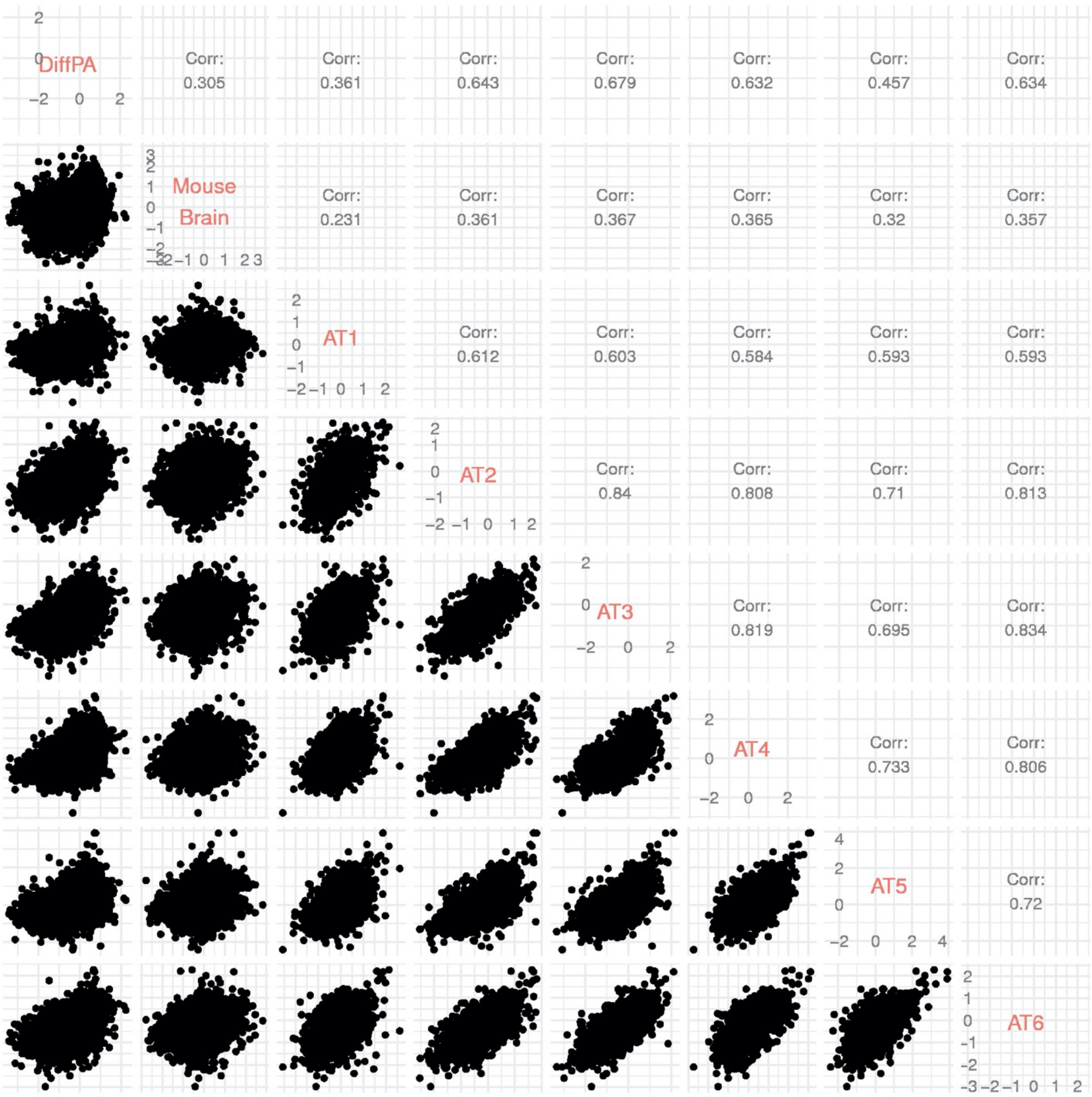


**Figure S3. Preservation of differential RNA profiles between nuclear-enriched and background-enriched droplets.**

Correlation plots of log fold changes across different snRNA-seq experiments. For each of the 8 experiments (differentiating preadipocytes (DiffPA), mouse brain, and six human frozen adipose tissue (AT) snRNA-seq samples), the log2 fold change of the counts per million (CPM) for each gene is calculated between the nuclear-enriched and background-enriched droplets. Nuclear-

enriched and background-enriched droplets are those with UMI counts greater than or equal to, and less than 100 UMI counts, respectively.

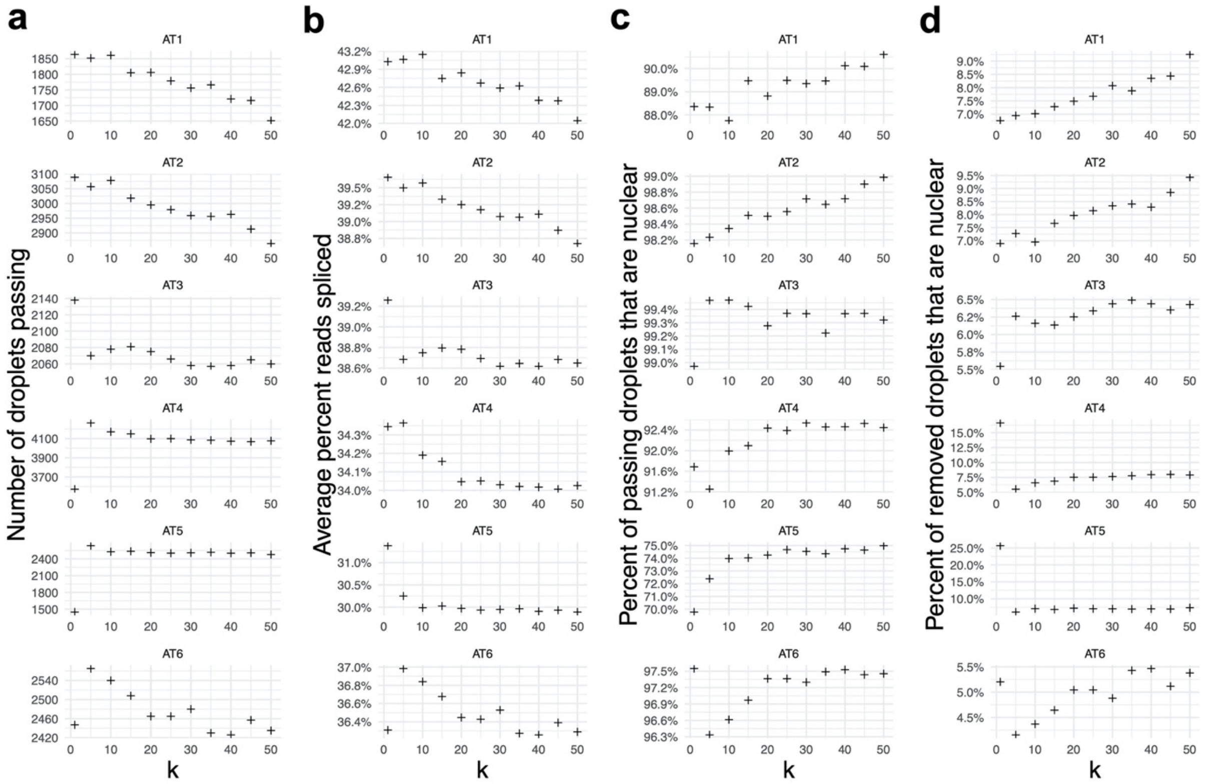


**Figure S4. Effect of the number of clusters on classification accuracy in a mixture model.** The number of clusters k was varied across values of 1, 5, …, 50 in the six adipose tissue samples. DIEM was run for each indicated k using a threshold value *t* of 0.5. The **(a)** number of droplets passing filtering and with a number of genes detected of at least 200, **(b)** the average percent of reads spliced, **(c)** the percent of passing droplets that are nuclear, and **(d)** the percent of removed droplets that are nuclear are shown. Nuclear droplets are defined as those with a percent of spliced reads below the sample-specific midpoint. Background and nuclear droplets are defined using the percent spliced reads.

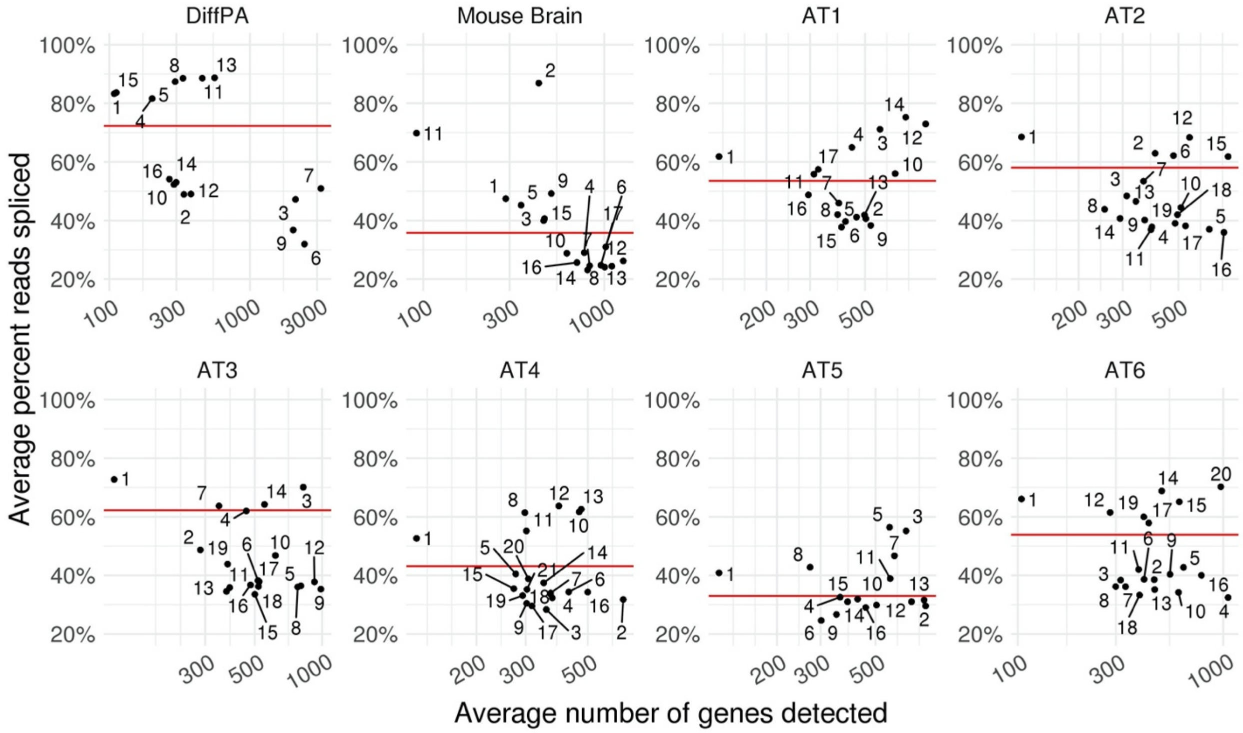


**Figure S5. Single-nucleus RNA-seq produces clusters with high levels of background contamination.**

The clusters produced by DIEM for the differentiating preadipocytes (DiffPA), mouse brain, and human frozen adipose tissue (AT) data sets are shown. Cluster 1 corresponds to the fixed debris cluster. The average number of genes detected in a cluster is plotted against the average percent of reads spliced. As each sample demonstrated a distinct distribution of spliced reads, we estimated a cutoff that separates nuclear and background droplets (see methods) for each sample (horizontal line). Clusters above and below the line indicate the background and nuclear clusters, respectively. This shows that clusters with high numbers of genes are susceptible to contamination.

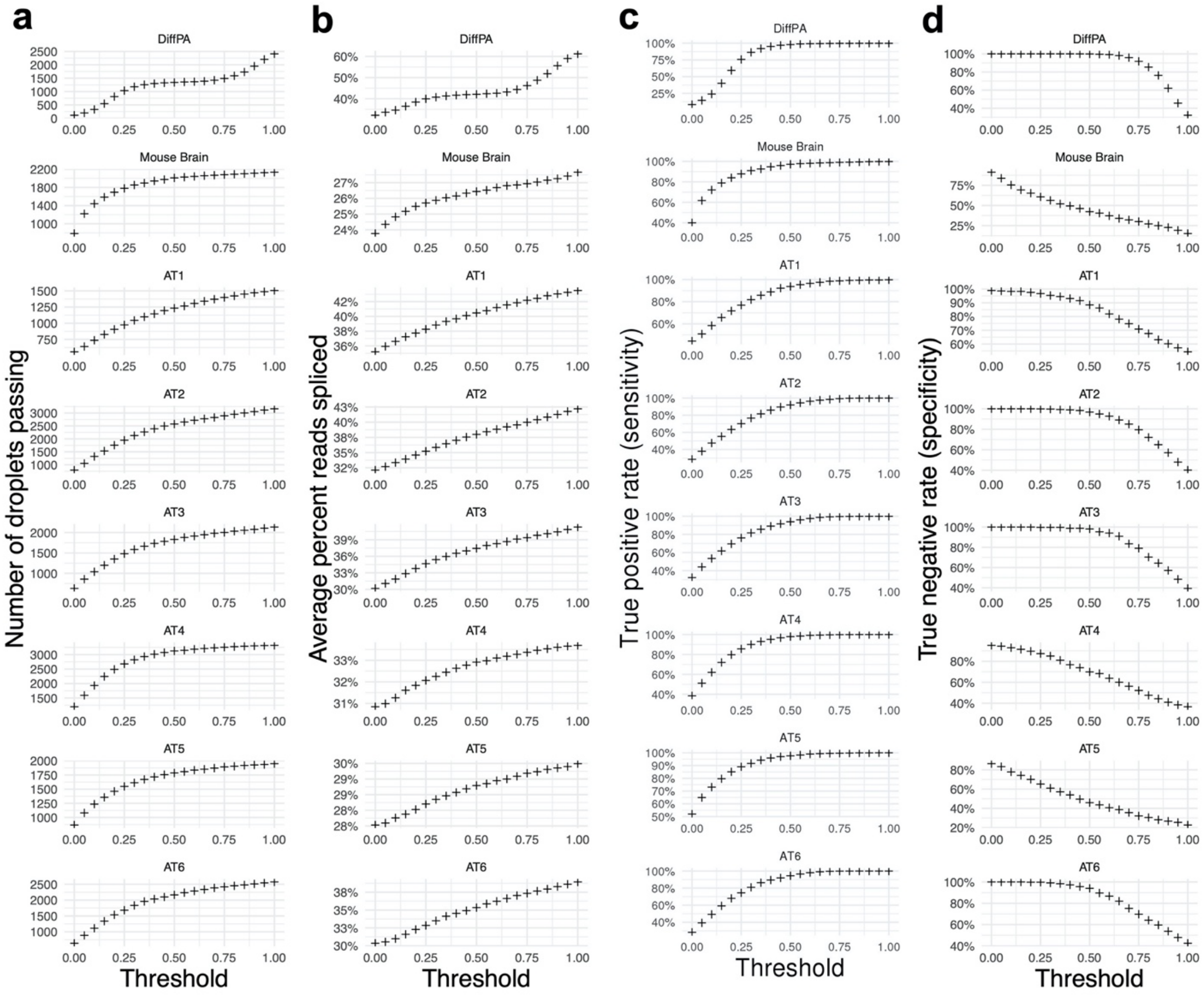

**Figure S6. Increasing the threshold parameter *t* increases sensitivity and decreases specificity.**

The figure shows the effect of varying the threshold parameter *t* from 0 to 1. DIEM was run on the differentiating preadipocytes (DiffPA), mouse brain, and human frozen adipose tissue (AT) data sets using k=20 clusters. **a,** The number of droplets that pass filtering and with a number of genes detected of at least 200. **b,** The average percent of reads spliced in droplets that pass DIEM filtering. **c,d,** The true positive and true negative rates are calculated for droplets with at least 200 genes detected. Nuclear and background droplets are defined as those with a percent of spliced reads below and above the sample-specific midpoint. **c,** The true positive rate (sensitivity), calculated as the percent of all nuclear droplets that correctly pass filtering, is

plotted against the threshold value. **d,** The true negative rate (specificity), calculated as the percent of all background droplets that are correctly removed, is plotted against the threshold value.

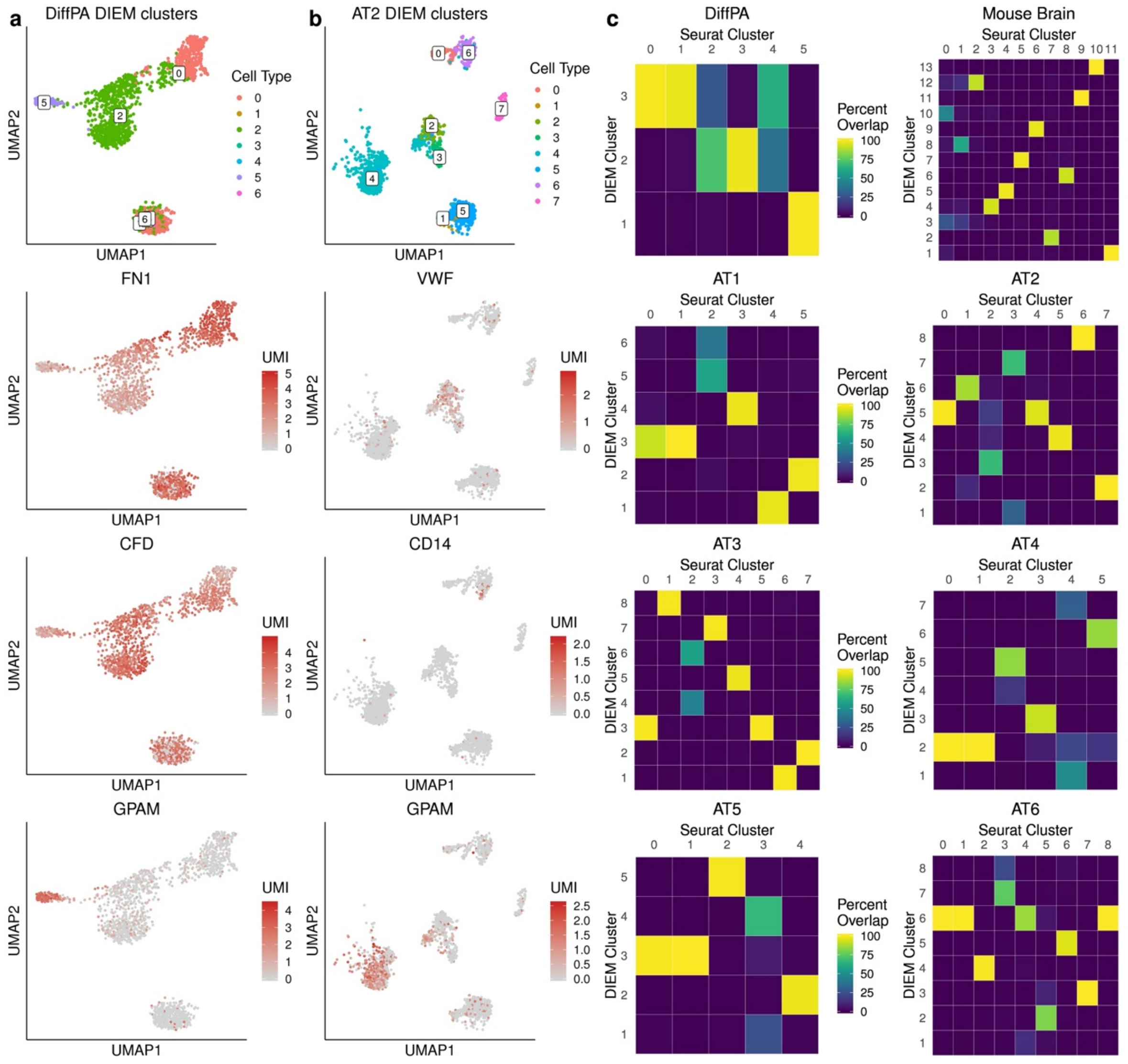


**Figure S7. Accurate modeling of major cell types by the multinomial mixture model in DIEM.**

**a,b,** UMAP[33] visualizations of clustering results after applying DIEM filtering for the **(a)** differentiating preadipocytes (DiffPA), and **(b)** adipose tissue sample 2 (AT2). The top panel shows the clusters identified by Seurat[20], while the bottom clusters show cell type marker expression in these clusters. The DiffPA data set consists of preadipocytes (expressing *CFD*), fibroblasts (expressing *FN1*), and adipocytes (expressing *GPAM*), while the AT consists of adipocyte (expressing *GPAM*), immune (expressing *CD14*), endothelial (expressing *VWF*), and

stromal cell types. **c,** Overlap of clusters identified by the DIEM mixture model with those from Seurat. Each panel shows the results from one of the eight independent data sets. The rows of the heatmap correspond to clusters identified by DIEM, while the columns correspond to Seurat clusters. Brighter values indicate a higher overlap. The percent overlap is defined as the number of shared droplets divided by the minimum size of the clusters in the pair. The average overlap was 73.0% across corresponding clusters for the DIEM clusters.

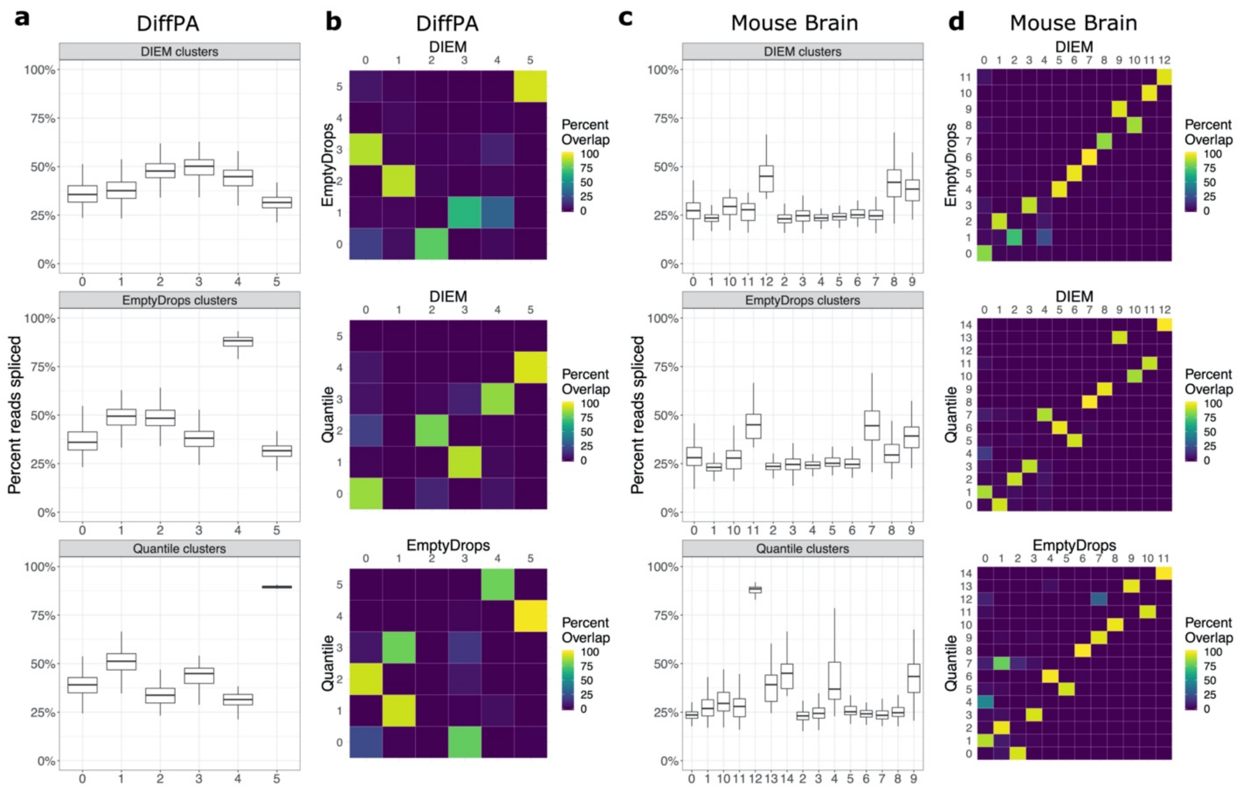


**Figure S8. DIEM filtering reduces contamination in clusters in the differentiating pre-adipocyte and mouse brain single-nucleus RNA-seq experiments.**

**a,b,** The **(a)** distribution of the percent of reads spliced for droplets in clusters identified by Seurat[20] after filtering with each of the three methods in the differentiating preadipocytes (DiffPA) is shown in a box plot. The **(b)** overlap of the resulting DiffPA clusters between the three filtering methods is shown in a heatmap. **c,d,** The **(c)** distribution of the percent of reads spliced for droplets in Seurat clustering after filtering with each of the three methods in the mouse brain is shown in a box plot. The **(d)** overlap of the resulting mouse brain clusters between the three filtering methods is shown in a heatmap. Brighter values in the heatmap indicate a higher percent overlap between the methods. Major cell types are preserved across the filtering methods.

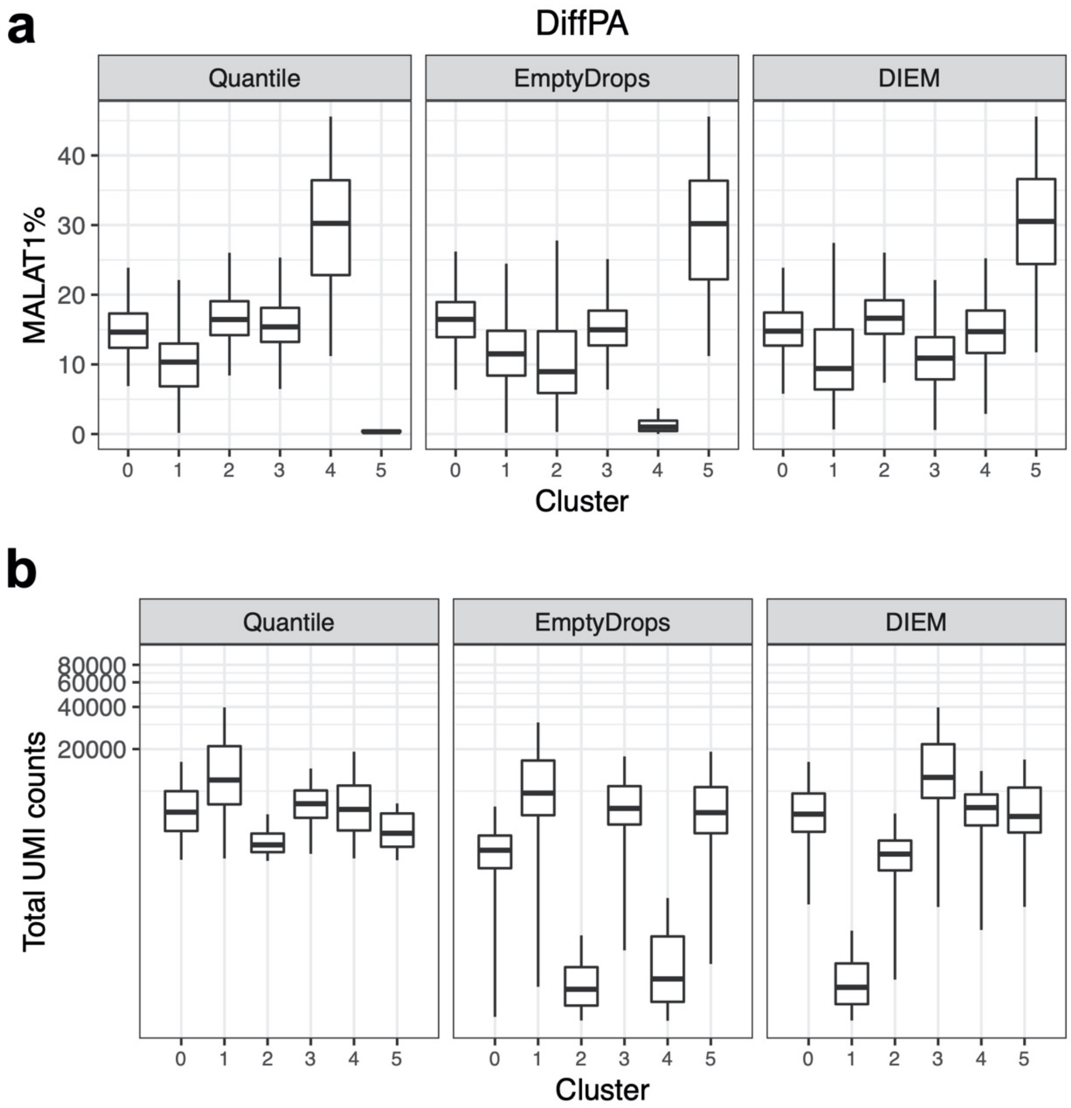


**Figure S9. DIEM removes clusters with high MALAT1 expression and is able to keep clusters with low read counts in the DiffPA snRNA-seq data set.**

**a,** Boxplots showing the percent of UMIs mapping to *MALAT1* (MALAT1%) per droplet in the differentiating preadipocytes (DiffPA). MALAT1% of clusters are compared across the quantile-based, EmptyDrops[12], and DIEM filtering methods. *MALAT1* is a nuclear-localized lincRNA[18], which suggests that the RNA is of nuclear origin. **b,** Boxplots showing the total number of UMIs

per droplet in the differentiating preadipocytes (DiffPA). Clusters are compared across the three filtering methods.

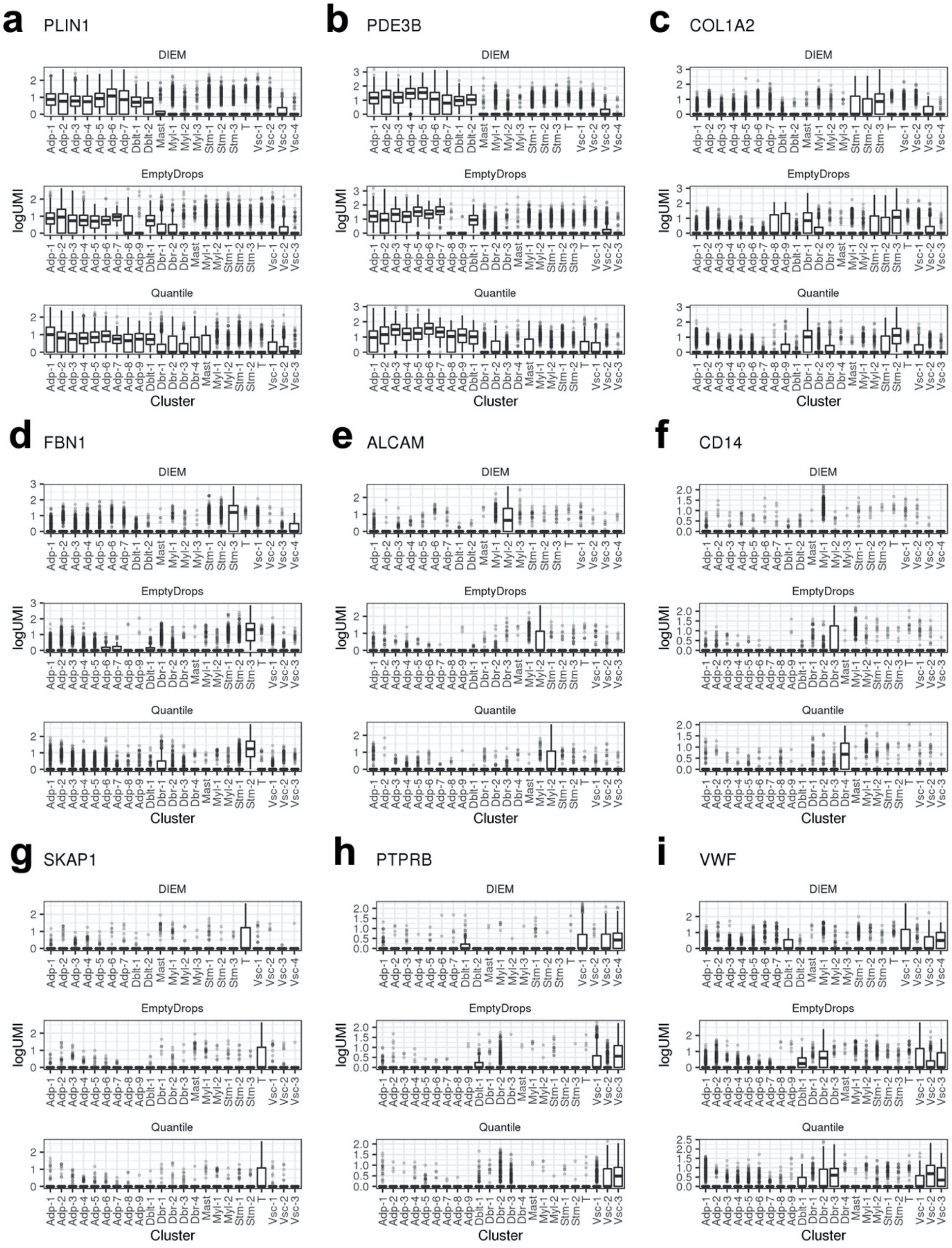

a PLIN1
b PDE3B
c COL1A2
d FBN1
e ALCAM
f CD14
g SKAP1
h PTPRB
i VWF
DIEM
EmptyDrops
Quantile
logUMI
Cluster

**Figure S10. Marker genes for major adipose tissue cell types identified after filtering and clustering.**

Clustering of droplets in the adipose tissue after filtering with DIEM, EmptyDrops[12], and the quantile method reveals adipose cell types. Droplets are clustered with Seurat[20] and unique molecular index (UMI) counts are normalized according to depth and then log-transformed. The expression of the cell type markers for **(a,b)** adipocyte, **(c,d)** stromal, **(e,f)** myeloid, **(g)** T cell, and **(h,i)** endothelial are shown for each cluster/cell type.

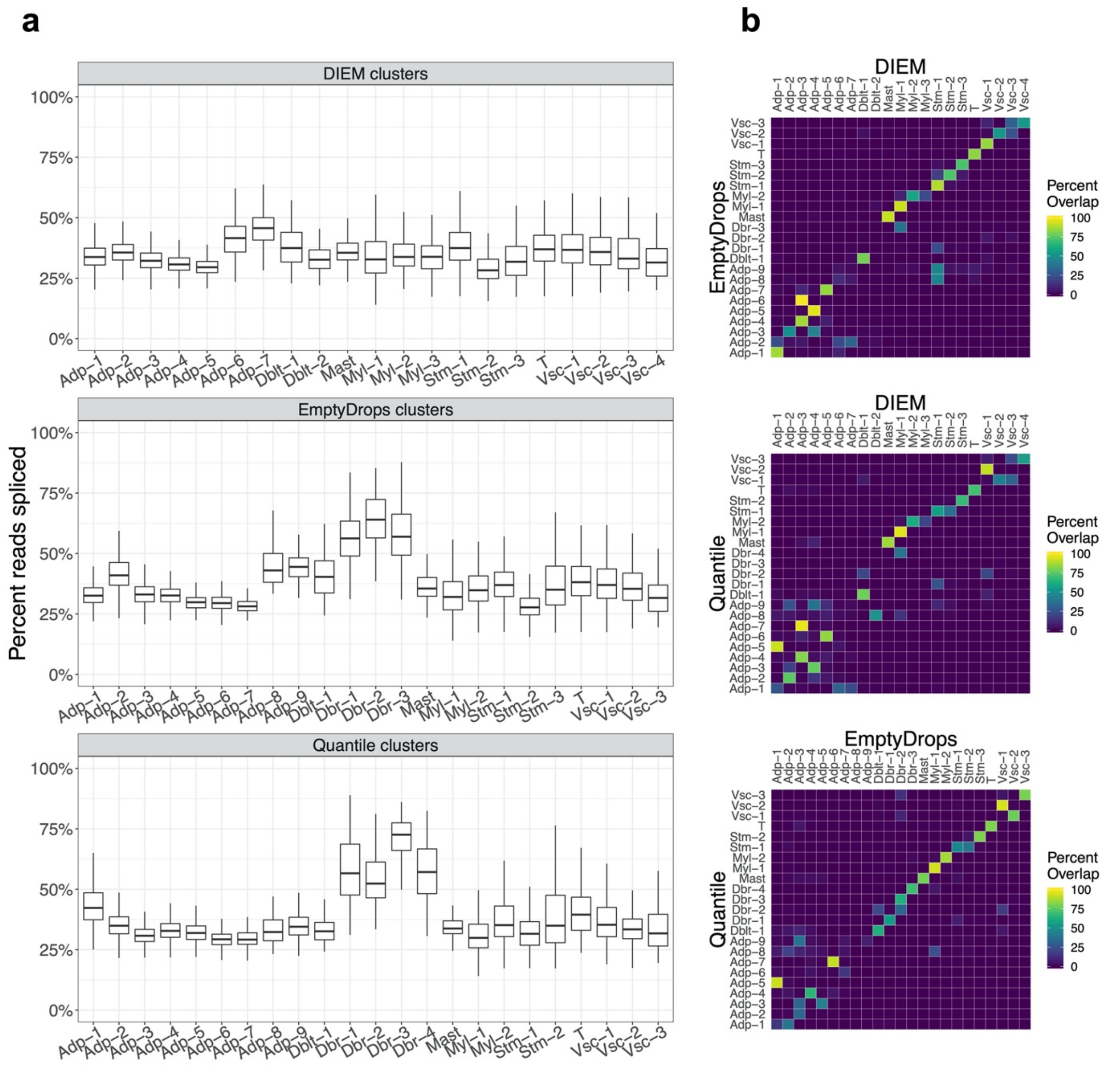


**Figure S11. DIEM filtering reduces contamination in clusters in the combined human adipose tissue single-nucleus RNA-seq experiments.**

**a,b,** The **(a)** distribution of the percent of reads spliced for droplets in Seurat[20] clusters after filtering with each of the three methods in the human adipose tissue is shown in a box plot. The **(b)** overlap of the resulting adipose tissue clusters between the three filtering methods is shown in a heatmap. The clusters/cell types correspond to adipocyte (Adp), doublet, (Dblt), debris (Dbr), mast, stromal (Stm), and vascular (Vsc). A cluster was classified as debris (Dbr) if it had a mean percent of spliced reads above 50%.

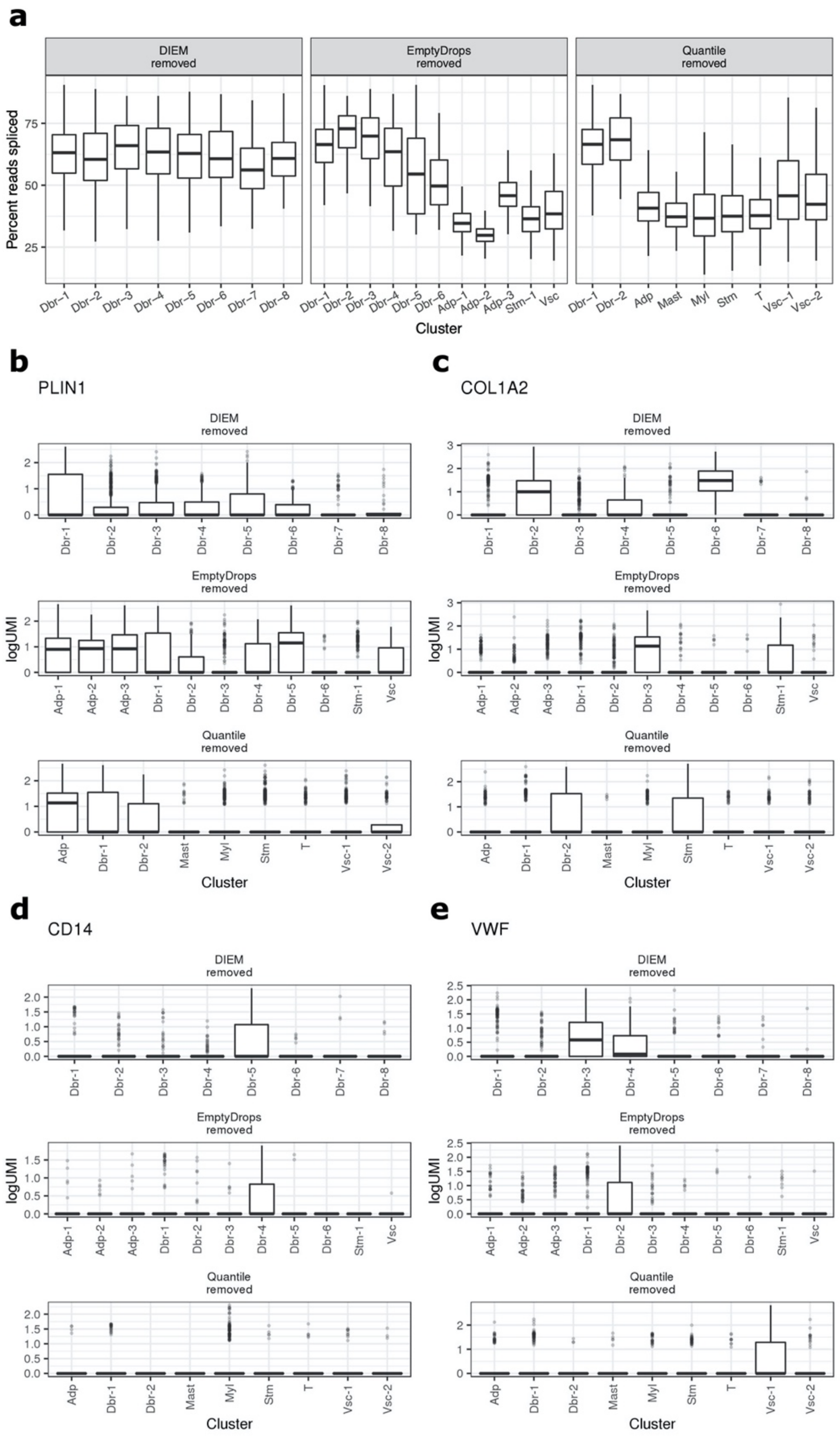


**Figure S12. Clustering of removed droplets shows cell type characteristics despite increased background RNA contamination.**

**a,** The distribution of percent of reads spliced in clusters formed by removed droplets in the adipose tissue. All clusters formed by DIEM-removed droplets consisted mostly of debris contaminated droplets (percent reads spliced > 50%). Of the 11 clusters formed by droplets removed by EmptyDrops[12], 7 were nuclear (percent of reads spliced > 50%). The droplets removed by the quantile-based approach formed 9 clusters, of which 7 were nuclear (percent of reads spliced > 50%). **b,c,d,e** The expression of marker genes for **(b)** adipocyte, **(c)** stromal, **(d)** macrophage, and **(e)** endothelial cell types are shown. Clustering was performed by Seurat[20] and unique molecular index (UMI) counts were normalized according to depth and then log-transformed. The clusters/cell types correspond to adipocyte (Adp), debris (Dbr), mast, stromal (Stm), and vascular (Vsc).

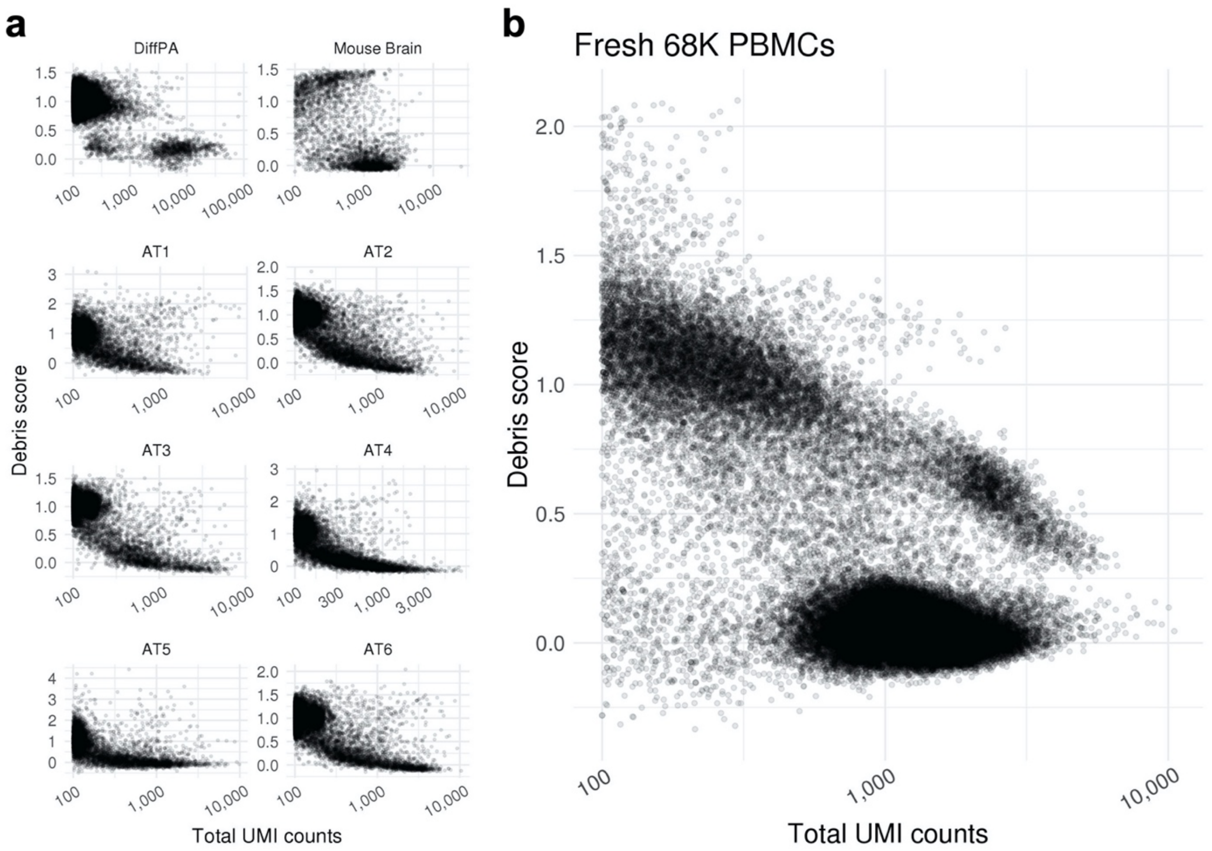


**Figure S13. Debris scoring is less effective in discriminating cell and ambient RNA droplets in single-cell RNA-seq**

**a,b,** Relationship between total UMI counts and debris score in droplets from the **(a)** snRNA-seq experiments and the **(b)** single-cell RNA-seq experiments. DIEM was run using k=20 and t=0.5 on the differentiating preadipocytes (DiffPA), mouse brain, 6 adipose tissue (AT) samples snRNA-seq data sets, as well as the peripheral blood mononuclear cells (PBMC) single-cell RNA-seq data set. The debris score shows a high specificity to discriminate debris from cell types in snRNA-seq, whereas it shows less specificity in the single-cell RNA-seq experiment.

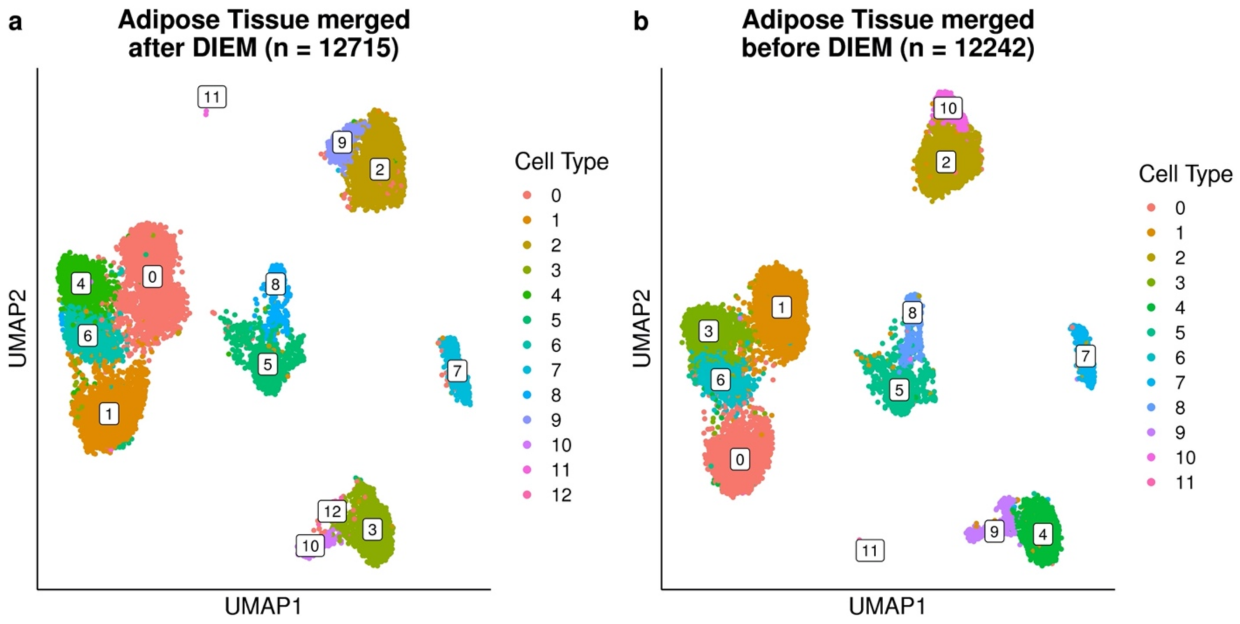


**Figure S14. Comparison of running DIEM filtering on the six adipose tissue samples individually or merged.**

**a,b,** The six adipose tissue samples were either **(a)** filtered using DIEM on each of the six samples and then combined, or **(b)** combined and then filtered using DIEM in the human frozen adipose tissue snRNA-seq data. Both approaches produced similar cell types, with the pre-merged data resulting in one less myeloid cell type.
